# Toxicity profiles of ROS1 tyrosine kinase inhibitors in advanced non-small cell lung cancer: a systematic review and proportional meta-analysis

**DOI:** 10.3389/fphar.2025.1644034

**Published:** 2025-08-29

**Authors:** Bo-Xuan Jiang, Jia-Wei Zeng, Jia-Jia Yan, Li-Yan Zhao

**Affiliations:** Department of Pharmacy, The First Affiliated Hospital of Sun Yat-sen University, Guangzhou, China

**Keywords:** ROS1, tyrosine kinase inhibitors, toxicity, non-small cell lung cancer, proportional meta-analysis

## Abstract

ROS1 tyrosine kinase inhibitors (TKIs) have shown significant efficacy in advanced ROS1-rearranged non-small cell lung cancer (NSCLC). However, no systematic investigation has been conducted on the toxicity profiles of these TKIs, which are critical for clinical decision-making and patient management. We conducted a systematic search across PubMed, Embase, the Cochrane Library, and ClinicalTrials.gov to identify studies that reported on the safety profiles of ROS1-TKIs in patients with advanced NSCLC. Eligible studies were those published between 1 January 2013 and 28 February 2025 in English language. A proportional meta-analysis was performed. Primary outcomes included the incidence rates of systemic all-grade adverse events (AEs; grades 1–5) and serious adverse events (SAEs; grades 3–5) for each ROS1-TKI, while secondary outcomes focused on incidence rates of specific AEs and SAEs. This systematic review and proportional meta-analysis included 26 studies involving 5,273 patients. ROS1-TKIs demonstrated high incidences of systemic all-grade AEs, ranging from 90% to 99%. Systemic SAEs exhibited greater variability across agents, ranging from 29% to 47%: crizotinib, 43% (95% CI, 36%–49%); ceritinib, 41% (95% CI, 37%–45%); lorlatinib, 39% (95% CI, 25%–55%); entrectinib, 32% (95% CI, 28%–36%); repotrectinib, 29% (95% CI, 24%–33%); iruplinalkib, 44% (95% CI, 38%–50%); and unecritinib, 47% (95% CI, 38%–56%). This indicated that repotrectinib might be more tolerable, while unecitinib might have a lower safety profile. Additionally, specific AE profiles varied across ROS1-TKIs: repotrectinib exhibited higher rates of dizziness, entrectinib demonstrated frequent fatigue, and lorlatinib showed an increased incidence of edema. Taletrectinib and unecritinib were notably associated with hepatotoxicity. This study presents the first comprehensive evaluation of ROS1-TKIs’ toxicity profiles in NSCLC patients. These findings will guide drug selection and safety monitoring, emphasizing the necessity of considering patients’ health status, potential risk factors, and the characteristics of ROS1-TKI-related adverse reactions.

**Systematic review registration:**

https://www.crd.york.ac.uk/PROSPERO/view/CRD42024551353, identifier CRD42024551353.

## 1 Introduction

According to the latest global cancer statistics, lung cancer remains the most prevalent malignancy and primary contributor to cancer-related mortality worldwide (Ferlay et al., 2024). Non-small cell lung cancer (NSCLC), representing approximately 85% of all lung cancer cases (Ganti et al., 2021), presents significant therapeutic challenges. The ROS proto-oncogene 1 (ROS1), an orphan receptor tyrosine kinase, plays a critical role in cellular differentiation, proliferation, growth, and survival through its fusion protein formations (Gendarme et al., 2022). Clinically relevant ROS1 gene rearrangements are identified in 0.9%–2.6% of NSCLC cases (Davies and Doebele, 2013). The application of ROS1 tyrosine kinase inhibitors (TKIs) has revolutionized treatment outcomes for advanced ROS1-rearranged NSCLC. Current clinical guidelines from the National Comprehensive Cancer Network (NCCN) recommend entrectinib, crizotinib, repotrectinib, ceritinib, and lorlatinib as first-line or subsequent therapies for advanced ROS1-rearranged NSCLC (Network, 2024). Notably, the Chinese National Medical Products Administration (NMPA) has approved unecritinib and taletrectinib specifically for ROS1-rearranged advanced NSCLC. Additionally, iruplinalkib, a novel highly selective ALK/ROS1 dual inhibitor, has demonstrated promising therapeutic potential in patients with ROS1-rearranged NSCLC in a phase I clinical trial (Shi et al., 2022).

While demonstrating significant clinical efficacy, ROS1-TKIs are associated with a spectrum of adverse events (AEs) that require vigilant monitoring and proactive management. Crizotinib is associated with a higher incidence of visual effects (60%–80%) and gastrointestinal AEs (Shaw et al., 2014), while entrectinib shows increased rates of cognitive impairment (10%–15%) and weight gain (Drilon et al., 2020). Lorlatinib frequently induces hypercholesterolemia (94%), edema (51%), and central nervous system effects (e.g., headache, mood changes) (Lu et al., 2022). Repotrectinib, a newer macrocyclic inhibitor, demonstrates improved tolerability but retains risks of dizziness (58%), dysgeusia (50%), and paresthesia (30%) (Drilon et al., 2024). The toxicity profiles of ROS1-TKIs exhibit substantial heterogeneity across different drugs. Current understanding of AE types and incidence rates is predominantly derived from individual clinical trials, which exhibit significant heterogeneity in study design parameters, including patient demographics, sample sizes, and follow-up durations.

To achieve a more precise and holistic understanding of the toxicity spectrum of ROS1-TKIs, we conducted a proportional meta-analysis to synthesize safety data from phase II/III single-arm studies and RCTs, presenting the range, incidence rate and severity of AEs associated with ROS1-TKIs. Our work provides high-level evidence for the safety management of individualized treatment with ROS1-TKIs, in order to facilitate the optimization of clinical decision-making for patients with ROS1-rearranged NSCLC.

## 2 Materials and methods

We performed a systematic review and proportional meta-analysis in accordance with the Preferred Reporting Items for Systematic Reviews and Meta-Analyses (PRISMA) guidelines and followed methodological standards outlined in the Cochrane Handbook for Systematic Reviews of Interventions. A prespecified protocol for this meta-analysis was registered on the PROSPERO platform (registration number: CRD42024551353).

### 2.1 Search strategies and study selection

We conducted a systematic search across three electronic databases (PubMed, Embase, and Cochrane Library) and ClinicalTrials.gov for studies published in English between 1 January 2013, and 28 February 2025. The detailed search strategies are provided in Supplementary Table S1. Study selection was performed by two independent reviewers (B-X.J and J-W.Z) according to the inclusion/exclusion criteria, discrepancies were resolved through consensus discussions with a third reviewer (L-Y.Z). The inclusion criteria were as follows: 1 studies involving patients with locally advanced or metastatic NSCLC; 2 at least one treatment group receiving ROS1-TKI monotherapy, including crizotinib, entrectinib, repotrectinib, lorlatinib, ceritinib, unecritinib, taletrectinib, and iruplinalkib; 3 phase II/III randomized controlled trials (RCTs) or phase II/III single-arm trials; and 4 AEs data reported according to the Common Terminology Criteria for Adverse Events (CTCAE). Studies were excluded if they met any of the following criteria: 1 conference abstracts without full-text availability; 2 AE data not comprehensively documented; or 3 trials with safety outcomes subsequently updated in publications with more mature or longer follow-up data.

### 2.2 Outcome measures and data extraction

Primary outcomes included the incidence rates of systemic all-grade AEs (grades 1–5) and serious adverse events (SAEs; grades 3–5) for each ROS1-TKI, reflecting the frequency and severity of toxicity, respectively. Secondary outcomes focused on incidence rates of specific AEs and SAEs. Notably, systemic AEs have a widespread, body-wide impact, while specific AEs are localized to particular organs, tissues, or systems. While treatment-related AEs (TRAEs) are clinically significant, most included studies reported TRAEs. To maintain analytical consistency, we prioritized TRAEs over all-cause AEs. Study ID, first author, year of publication, clinical trial phase, study design, treatment regimen, sample size, number of adverse events and patient characteristics were extracted. Data were extracted by two independent reviewers (B-X.J and J-W.Z) and any discrepancies were settled by consensus.

### 2.3 Quality assessment

Two independent reviewers (B-X.J and J-W.Z) evaluated the methodological quality of the included studies. Discrepancies in assessments were resolved through consensus discussions with a third reviewer (L-Y.Z). The risk of bias in single-arm studies was analyzed using the ROBINS-I V2 tool, while RCTs were assessed via RoB 2 tool (Sterne et al., 2019). Risk-of-bias visualizations of RCTs were generated using Review Manager (version 5.3).

### 2.4 Data synthesis and statistical analysis

STATA (MP version 17.0) was used for all analyses. Incidences of AEs were presented as mean values with 95% confidence intervals (CIs). A random-effects model was utilized to synthesize the summary incidences of both systemic and specific AEs/SAEs due to inter-study heterogeneity. Statistical analyses, including data pooling and forest plot generation were conducted through the *metaprop* package in Stata, which was not affected by data characteristics. When some studies reported an incidence of 0% or 100%, *metaprop* could still produce reasonable results. Heterogeneity across studies was quantified using the *I* ^2^ statistic derived from the Cochrane *Q*-test, with values exceeding 50% indicating significant heterogeneity (Higgins JP et al., 2003). Subgroup analyses via *Z*-tests were conducted within a stratified analytic framework incorporating clinically relevant variables: gender distribution, ethnic composition, age, publication date, and study design. Sensitivity of this analysis was assessed by leave-one-out analysis, while publication bias was estimated using the Galbraith plot.

## 3 Results

### 3.1 Eligible studies and characteristics

A systematic search identified 1,761 potentially relevant studies, comprising 1,644 records from electronic databases and 117 from ClinicalTrials.gov. Following initial screening, 99 full-text articles underwent eligibility assessment. Of these, 73 were excluded due to predefined criteria outlined in Supplementary Table S2. Ultimately, 26 studies (15 single-arm trials and 11 RCTs) involving 5,273 patients met the inclusion criteria (Supplementary Figure S1). Among these studies, crizotinib was assessed in 2 single-arm trials (Blackhall et al., 2017; Wu et al., 2022) and 9 RCTs (Peters et al., 2017; Solomon et al., 2018b; Wu et al., 2018; Zhou et al., 2019; Camidge et al., 2020; Shaw et al., 2020; Horn et al., 2021; Shi et al., 2024), ceritinib in 5 single-arm trials (Crinò et al., 2016; Lim et al., 2017; Hida et al., 2018; Nishio et al., 2020) and 2 RCTs (Shaw et al., 2017; Soria J. C. et al., 2017), and lorlatinib in 3 single-arm trials (Solomon et al., 2018a; Seto et al., 2020; Lu et al., 2022) and 1 RCT (Shaw et al., 2020). Other agents showed fewer investigations: repotrectinib (Drilon et al., 2024), unecritinib (Lu et al., 2023) and taletrectinib (Li et al., 2024) each had 1 single-arm trial; iruplinalkib was investigated in 1 single-arm trial (Shi et al., 2023) and 1 RCT (Shi et al., 2024); AE data for entrectinib were only reported in an integrated analysis of single-arm trials (Dziadziuszko et al., 2021b). The comprehensive characteristics of these included studies are systematically presented in Table 1.

**TABLE 1:** Baseline characteristics of the included studies.

Study/Author, year	Phase	Study design	Intervention	Treatment	Patients(n)	Female(n)	Median age(y)	Median follow-up months	PS 0-1 (%)	Nonsmoker (%)	Ethnicity
PROFILE 1005, 2017 (Blackhall et al., 2017)	II	Single-arm	Crizotinib	250 mg BID	1066	601	52.2	45.6	84	66	Multiple
Wu YL, 2022 (Wu et al., 2022)	II	Single-arm	Crizotinib	250 mg BID	127	73	52.48	56.1	100	71.7	Multiple
PROFILE 1029, 2018 (Wu et al., 2018)	III	RCT	Crizotinib	250 mg BID	104	54	48.2	22.5	96.2	75	Multiple
PROFILE 1007, 2014 (Pfizer, 2017)	III	RCT	Chemotherapy	250 mg BID	103	60	48.9	21.6	96.1	69.9	Multiple
			Crizotinib		173	98	50.3	32.1	89	62	—
			Chemotherapy		174	95	49.8	24.4	91	64	—
PROFILE 1014, 2018 (Solomon et al., 2018b)	III	RCT	Crizotinib	250 mg BID	172	104	50.9	36	76.8	88.8	Multiple
			Chemotherapy		171	108	52.9				Multiple
ALESIA, 2019 (Zhou et al., 2019)	III	RCT	Crizotinib	250 mg BID	62	28	51.1	15.0	98	73	Asia
			Alectinib	600 mg BID	125	61	50.5	16.2	97	67	Asia
ALEX, 2017 (Peters et al., 2017)	III	RCT	Crizotinib	250 mg BID	151	87	53.8	17.6	93	65	Multiple
			Alectinib	600 mg BID	152	84	56.3	18.6	93	61	Multiple
ALTA-1L, 2020 (Camidge et al., 2020)	III	RCT	Crizotinib	250 mg BID	138	81	58.6	15.2	100	54.3	Multiple
			Brigatinib	250 mg BID	137	69	57.9	40.4	100	61.3	Multiple
eXalt, 2021 (Horn et al., 2021)	III	RCT	Crizotinib	250 mg BID	147	70	53.0	20.2	95.2	63.9	—
			Ensartinib	250 mg BID	143	71	54.0	23.8	95.1	59.4	—
CROWN, 2020 (Shaw et al., 2020)	III	RCT	Crizotinib	250 mg BID	147	91	55.6	14.8	94	64	Multiple
			Lorlatinib	100 mg QD	149	84	59.1	18.3	98	54	Multiple
ASCEND-2, 2016 (Crinò et al., 2016)	II	Single-arm	Ceritinib	750 mg QD	140	70	51.2	11.3	85.7	—	Multiple
ASCEND-3, 2019 (Nishio et al., 2020)	II	Single-arm	Ceritinib	750 mg QD	124	74	54.8	52.1	92.7	—	Multiple
ASCEND-9, 2018 (Hida et al., 2018)	II	Single-arm	Ceritinib	750 mg QD	20	12	52.2	11.6	100	40	Asian
NCT02040870 (Pharmaceuticals, 2019)	I/II	Single-arm	Ceritinib	750 mg QD	103	48	49.3	7.5	—	—	Asian
^δ^Lim SM, 2017 (Lim et al., 2017)	II	Single-arm	Ceritinib	750 mg QD	32	24	62.0	14.0	88	84	Asian
ASCEND-5, 2017 (Shaw et al., 2017)	III	RCT	Ceritinib	750 mg QD	115	68	53.1	16.6	92	62	Multiple
			Chemotherapy		116	61	54.4	16.4	96	53	Multiple
ASCEND-4, 2017 (Soria et al., 2017a)	III	RCT	Ceritinib	750 mg QD	189	102	54.5	23.9	94	57	Multiple
			Chemotherapy		187	114	53.3	11.1	92	65	Multiple
^δ^Solomon BJ, 2018 (Solomon et al., 2018a)	I/II	Single-arm	Lorlatinib	100 mg QD	275	157	54.0	6.9	96	—	Multiple
Lu S, 2022 (Lu et al., 2022)	II	Single-arm	Lorlatinib	100 mg QD	109	—	51.0	11.3	95.5	63.3	Asian
Seto T, 2020 (Seto et al., 2020)	II	Single-arm	Lorlatinib	100 mg QD	39	21	52.2	11.1	100	—	Asian
D.Rafal, 2021 (Dziadziuszko et al., 2021a)	I/II	Single-arm	Entrectinib	600 mg QD	504	—	—	15.8	90.1	62.7	Multiple
TRIDENT-1, 2021 (Drilon et al., 2024)	I/II	Single-arm	Repotrectinib	160 mg BID	426	—	—	24.0	100	63.8	Multiple
^δ^TRUST-I, 2024 (Li et al., 2024)	II	Single-arm	Taletrectinib	600 mg QD	173	100	55.0	23.5	100	73.4	—
INSPIRE, 2024 (Shi et al., 2024)	III	RCT	Crizotinib	250 mg BID	149	62	55.0	25.9	98.7	—	Asian
			Iruplinalkib	180 mg QD	143	72	55.0	26.7	99.3	—	Asian
INTELLECT, 2023 (Shi et al., 2023)	II	Single-arm	Iruplinalkib	180 mg QD	146	77	52.4	18.2	96.6	—	Asian
Lu S, 2023 (Lu et al., 2023)	I/II	Single-arm	Unecritinib	300 mg BID	150	88	52.0	20.3	100	72.1	Asian

Note. RCT: rondom controlled trial.

^δ^studies only reported event numbers of specific AEs/SAEs, not that of systemic AEs/SAEs.

The risk of bias of single-arm studies and RCTs is presented in Supplementary Table S3 and Supplementary Figure S2, respectively. Seven of the 15 single-arm studies were rated as high quality and 8 as medium quality. Assessments of RCTs identified limitations in the blinding of participants and personnel, which may introduce a high risk of bias, given that all included studies were open-label trials without built-in blinding procedures.

#### 3.2 Primary outcomes: incidences of systemic all-grade AEs and SAEs associated with ROS1-TKIs

**FIGURE 1:**
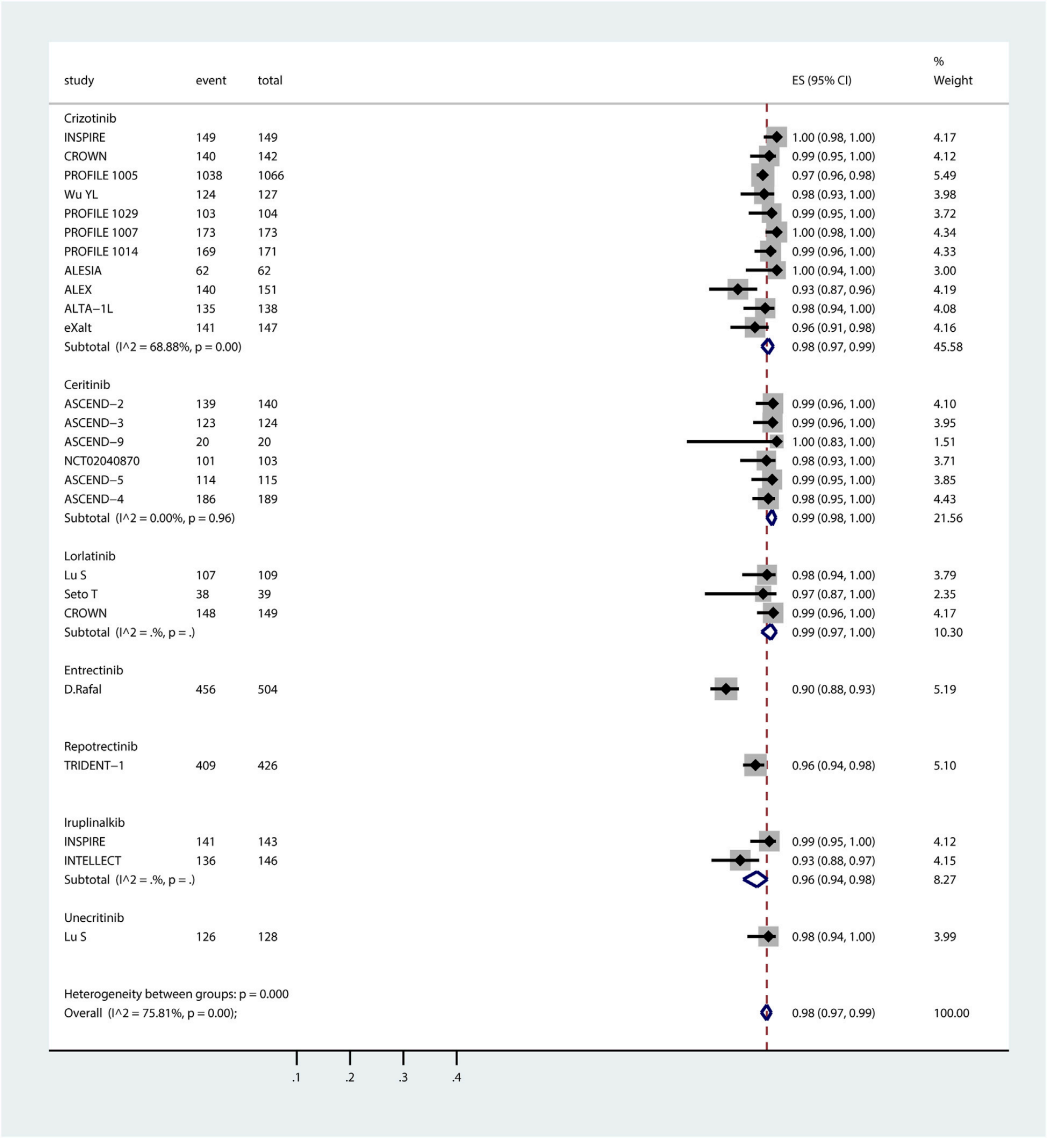
Forest plot of pooled incidence of systemic all-grade AEs associated with ROS1-TKIs via proportional meta-analysis. The ROS1-TKIs included crizotinib, ceritinib, lorlatinib, entrectinib, repotrectinib, iruplinalkib, and unecitinib. Taletrectinib was not included as the study on it did not report the incidence of systemic AEs. For each individual study included in the analysis, the plot presents the number of AE events, total participants, effect size (ES) with 95% CI for AE incidence, and the relative weight of the study in the meta-analysis. Subgroup analyses are stratified by TKI, with each subgroup displaying the pooled AE incidence (estimated incidence [95% CI]) alongside heterogeneity statistics (*I* ^*2*^ and p-value). The overall pooled incidence of systemic all-grade AEs across all ROS1-TKIs, accounting for all included studies, is summarized at the bottom of the plot.

**FIGURE 2:**
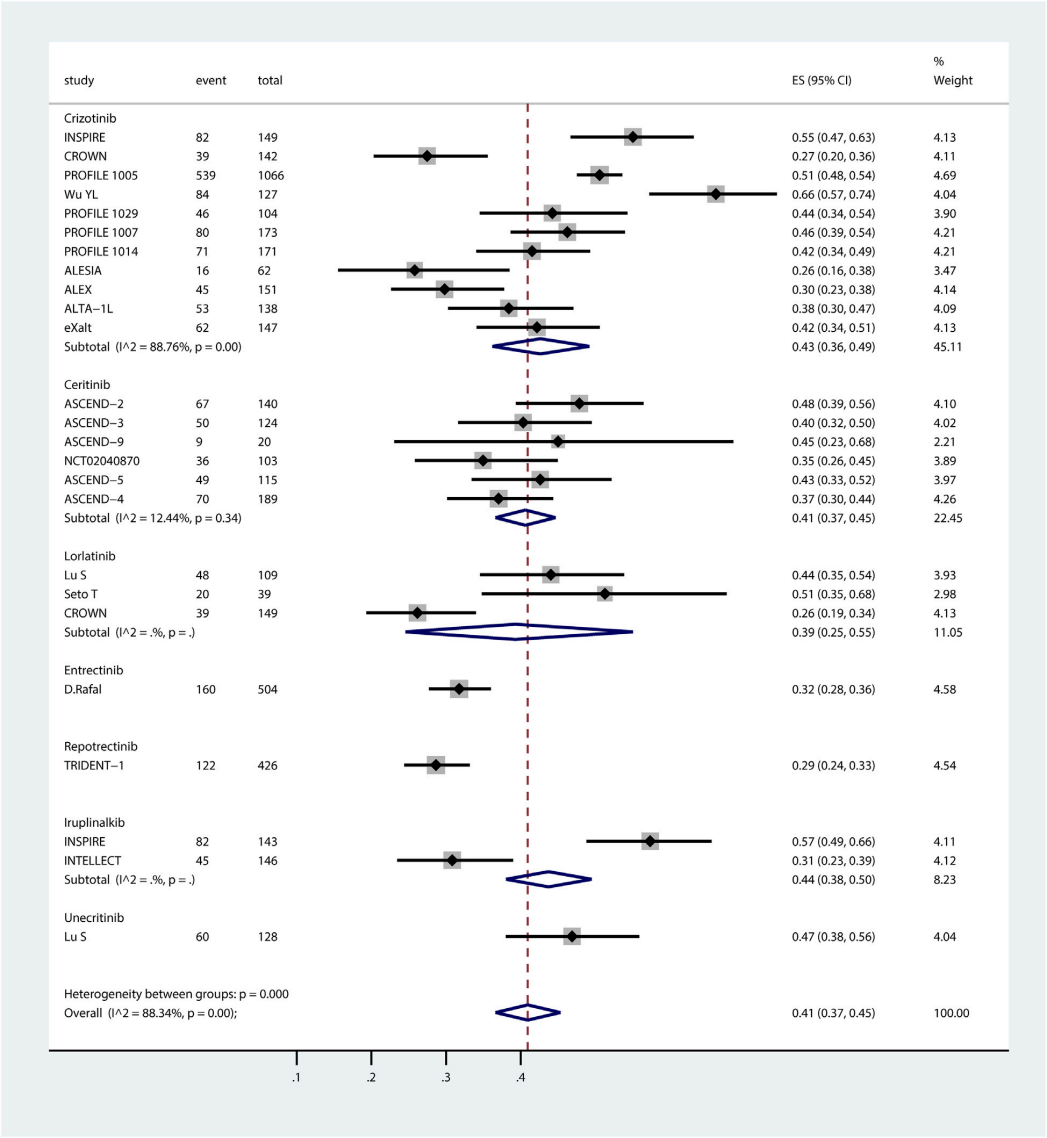
Forest plot of pooled incidence of systemic SAEs associated with ROS1-TKIs via proportional meta-analysis. The ROS1-TKIs included crizotinib, ceritinib, lorlatinib, entrectinib, repotrectinib, iruplinalkib, and unecitinib. Taletrectinib was not included as the study on it did not report the incidence of systemic SAEs. For each individual study included in the analysis, the plot presents the number of SAE events, total participants, effect size (ES) with 95% CI for SAE incidence, and the relative weight of the study in the meta-analysis. Subgroup analyses are stratified by TKI, with each subgroup displaying the pooled SAE incidence (estimated incidence [95% CI]) alongside heterogeneity statistics (*I* ^*2*^ and p-value). The overall pooled incidence of systemic SAEs across all ROS1-TKIs, accounting for all included studies, is summarized at the bottom of the plot.

Of the 26 studies identified, 3 studies (TRUST-I (Li et al., 2024), Solomon et al. (2018a) and Lim et al. (2017) were excluded due to not reporting the numbers of systemic AEs/SAEs. 23 studies were included in the proportional meta-analysis. The pooled incidence rates of systemic all-grade AEs and SAEs, derived via random-effects meta-analysis, are summarized in the forest plots in Figures 1, 2.

ROS1-TKIs demonstrated high incidences of systemic all-grade AEs, ranging from 90% to 99%. The summary incidence of each ROS1-TKI was as follows: crizotinib, 98% (95% CI, 97%–99%); ceritinib, 99% (95% CI, 98%–100%); lorlatinib, 99% (95% CI, 97%–100%); entrectinib, 90% (95% CI, 88%–93%); repotrectinib, 96% (95% CI, 94%–98%); iruplinalkib, 98% (95% CI, 94%–98%); and unecritinib, 98% (95% CI, 94%–100%). Systemic all-grade AEs and SAEs associated with taletrectinib were not reported here because systemic AE data were not documented in TRUST-I study (Li et al., 2024). Notably, the observation that the reported incidence of systemic AEs for entrectinib appears lower than other ROS1-TKIs may be due to the data being extraced from a pooled analysis of both phase I and phase II RCT studies. The absence of individual study data limits the ability to assess phase-specific safety signals and underrepresent the true safety profile of entrectinib, emphasizing the need for cautious cross-trial comparisons.

Systemic SAEs exhibited greater variability across agents, ranging from 29% to 47%. Pooled incidences were: crizotinib, 43% (95% CI, 36%–49%); ceritinib, 41% (95% CI, 37%–45%); lorlatinib, 39% (95% CI, 25%–55%); entrectinib, 32% (95% CI, 28%–36%); repotrectinib, 29% (95% CI, 24%–33%); iruplinalkib, 44% (95% CI, 38%–50%); and unecritinib, 47% (95% CI, 38%–56%). For ceritinib, trial dosages were typically higher than those in clinical practice, a factor that warrants attention when applying the findings of this study clinically.

#### 3.3 Secondary outcomes: incidence of specific all-grade AEs and SAEs associated with ROS1-TKIs

Twenty-six studies were included in the proportional meta-analysis of specific AEs/SAEs. The incidences of specific all-grade AEs, including rash, cough, dizziness, fatigue, edema, transaminase elevation (AST/ALT), gastrointestinal disturbances (diarrhea, vomiting, nausea, constipation), sinus bradycardia, hematological toxicities (anemia, neutropenia), and ocular disorders, were analyzed using a random-effects meta-analysis and are summarized in Table 2. The specific AE profiles varied significantly across ROS1-TKIs. Repotrectinib demonstrated a high incidence of dizziness (60%, 95% CI 55%–65%), while entrectinib was associated with fatigue (32%, 95% CI 28%–36%). Lorlatinib showed a higher incidence of edema (38%, 95% CI 30%–46%). Hepatotoxicity-related AEs were prominent with taletrectinib and unecritinib: taletrectinib exhibited elevated AST (76%, 95% CI 69%–82%) and ALT (68%, 95% CI 60%–75%), whereas unecritinib showed similarly high AST (73%, 95% CI 65%–81%) and ALT (72%, 95% CI 63%–79%) increases. Gastrointestinal AEs included diarrhea (crizotinib: 45%, 95% CI 32%–58%; ceritinib: 80%, 95% CI 76%–85%; taletrectinib: 70%, 95% CI 63%–77%), vomiting (ceritinib: 62%, 95% CI 57%–68%; taletrectinib: 53%, 95% CI 45%–61%; unecritinib: 60%, 95% CI 51%–69%), and nausea (ceritinib: 71%, 95% CI 63%–78%). Other notable AEs included constipation with crizotinib (39%, 95% CI 33%–47%), sinus bradycardia with unecritinib (47%, 95% CI 38%–56%), anemia with taletrectinib (49%, 95% CI 41%–57%), neutropenia with unecritinib (55%, 95% CI 46%–64%), and ocular disorders with crizotinib (32%, 95% CI 16%–52%).

**TABLE 2:** Pooled incidence of specific all-grade AEs of ROS1-TKIs via proportional meta-analysis.

All-grade AEs	Crizotinib	Ceritinib	Lorlatinib	Entrectinib	Repotrectinib	Taletrectinib	Iruplinalkib	Unecritinib
Systemic	0.98 [0.97–0.99]	0.99 [0.98–1.00]	0.99 [0.97–1.00]	0.90 [0.88–0.93]	0.96 [0.94–0.98]	—	0.96 [0.94–0.98]	0.98 [0.94–1.00]
Rash	0.08 [0.05–0.13]	0.13 [0.08–0.18]	0.03 [0.00–0.08]	0.06 [0.04–0.08]	—	—	0.18 [0.14–0.23]	0.11 [0.06–0.18]
Cough	0.14 [0.07–0.22]	0.18 [0.11–0.23]	0.02 [0.00–0.11]	—	—	—	—	—
Dizziness	0.14 [0.07–0.22]	0.11 [0.08–0.14]	0.03 [0.00–0.14]	0.27 [0.23–0.31]	0.60 [0.55–0.65]	0.23 [0.17–0.30]	—	0.11 [0.06–0.18]
Fatigue	0.16 [0.09–0.25]	0.29 [0.22–0.36]	0.04 [0.00–0.12]	0.32 [0.28–0.36]	0.17 [0.14–0.2]	—	—	0.19 [0.12–0.27]
Oedema	0.33 [0.25–0.42]	0.03 [0.00–0.07]	0.38 [0.30–0.46]	0.14 [0.11–0.17]	—	—	—	0.23 [0.16–0.31]
AST increased	0.29 [0.14–0.46]	0.46 [0.39–0.62]	0.19 [0.08–0.33]	0.13 [0.10–0.18]	0.18 [0.14–0.22]	0.76 [0.69–0.82]	0.52 [0.46–0.67]	0.73 [0.65–0.81]
ALT increased	0.37 [0.28–0.49]	0.51 [0.44–0.57]	0.21 [0.08–0.38]	0.12 [0.09–0.15]	0.18 [0.14–0.22]	0.68 [0.60–0.75]	0.44 [0.38–0.49]	0.72 [0.63–0.79]
Diarrhea	0.45 [0.32–0.58]	0.80 [0.76–0.85]	0.10 [0.02–0.24]	0.23 [0.19–0.27]	—	0.70 [0.63–0.77]	0.09 [0.06–0.13]	0.43 [0.34–0.52]
Vomiting	0.49 [0.41–0.56]	0.62 [0.57–0.68]	0.21 [0.00–0.80]	0.12 [0.09–0.15]	—	0.53 [0.45–0.61]	0.16 [0.12–0.20]	0.60 [0.51–0.69]
Nausea	0.44 [0.35–0.54]	0.71 [0.63–0.78]	0.04 [0.00–0.16]	0.20 [0.16–0.24]	0.12 [0.09–0.16]	0.42 [0.35–0.50]	0.18 [0.14–0.22]	0.36 [0.28–0.45]
Constipation	0.39 [0.33–0.47]	0.18 [0.10–0.27]	0.05 [0.00–0.20]	0.24 [0.20–0.28]	0.26 [0.22–0.30]	0.17 [0.12–0.24]	0.04 [0.02–0.06]	0.32 [0.24–0.41]
Sinus bradycardia	0.07 [0.02–0.15]	—	—	—	—	—	0.04 [0.02–0.06]	0.47 [0.38–0.56]
Anemia	0.11 [0.07–0.15]	0.12 [0.06–0.20]	0.09 [0.02–0.19]	0.14 [0.11–0.17]	0.30 [0.26–0.34]	0.49 [0.41–0.57]	0.05 [0.03–0.08]	0.26 [0.18–0.34]
Neutropenia	0.10 [0.04–0.18]	0.02 [0.00–0.05]	—	0.07 [0.05–0.09]	—	0.26 [0.20–0.33]	—	0.55 [0.46–0.64]
Eye disorders	0.32 [0.16–0.52]	—	—	0.08 [0.04–0.08]	—	—	—	0.28 [0.21–0.37]

Note. Incidences of AEs, were presented as mean values in decimal form with 95% confidence intervals (CIs); -: incidence of AE, was not documented in corresponding study; AST: aspartate aminotransferase; ALT: alanine aminotransferase.

SAEs reported in the included studies are detailed in Table 3. Taletrectinib and unecritinib also exhibited a higher incidence of hepatotoxicity-related SAEs, with taletrectinib-associated AST elevation (8%, 95% CI 4%–13%) and unecritinib-associated ALT elevation (8%, 95% CI 4%–14%) exceeding the 5% threshold. Other SAEs, including rash, dizziness, fatigue, gastrointestinal events, and anemia, occurred at lower frequencies (1%–5%).

**TABLE 3:** Pooled incidence of specific SAEs of ROS1-TKis via proportional meta-analysis.

SAEs	Crizotinib	Ceritinib	Lorlatinib	Entrectinib	Repotrectinib	Taletrectinib	Iruplinalkib	Unecritinib
Systemic	0.43 [0.36–0.49]	0.41 [0.37–0.45]	0.39 [0.25–0.55]	0.32 [0.28–0.36]	0.29 [0.24–0.33]	—	0.44 [0.38–0.50]	0.47 [0.38–0.56]
Rash	—	—	—	—	—	—	0.03 [0.01–0.08]	0.01 [0.00–0.04]
Dizziness	0.01 [0.00–0.01]	0.01 [0.00–0.01]	0.01 [0.00–0.01]	—	0.03 [0.01–0.05]	0.01 [0.00–0.03]	—	—
Fatigue	0.01 [0.00–0.01]	0.01 [0.00–0.01]	0.01 [0.00–0.01]	0.03 [0.02–0.05]	0.01 [0.00–0.02]	—	—	0.01 [0.01–0.04]
AST increased	0.01 [0.00–0.02]	0.01 [0.00–0.02]	0.01 [0.00–0.02]	0.02 [0.01–0.04]	0.01 [0.01–0.03]	0.08 [0.04–0.13]	0.03 [0.00–0.10]	0.04 [0.01–0.09]
ALT increased	0.02 [0.01–0.03]	0.01 [0.00–0.02]	0.01 [0.00–0.02]	0.02 [0.01–0.04]	0.01 [0.01–0.03]	0.05 [0.02–0.10]	0.03 [0.00–0.11]	0.08 [0.04–0.14]
Diarrhea	0.01 [0.00–0.01]	0.01 [0.00–0.03]	0.01 [0.00–0.01]	0.02 [0.01–0.04]	—	0.03 [0.01–0.07]	0.01 [0.00–0.02]	—
Vomiting	0.01 [0.00–0.01]	0.03 [0.02–0.04]	0.01 [0.00–0.02]	—	—	0.01 [0.00–0.03]	—	0.01 [0.00–0.04]
Nausea	0.01 [0.00–0.01]	0.01 [0.00–0.01]	—	—	0.01 [0.00–0.02]	0.01 [0.00–0.03]	—	—
Anemia	0.01 [0.00–0.02]	0.01 [0.00–0.01]	0.01 [0.00–0.01]	0.03 [0.02–0.05]	0.04 [0.02–0.08]	0.02 [0.00–0.05]	0.01 [0.00–0.02]	0.02 [0.00–0.06]

Note. Incidences of AEs, were presented as mean values in decimal form with 95% confidence intervals (CIs); -: incidence of AE, was not documented in corresponding study; AST: aspartate aminotransferase; ALT: alanine aminotransferase.

These findings highlight the heterogeneous safety profiles of ROS1-TKIs, underscoring the need for tailored monitoring, particularly for hepatotoxicity with taletrectinib and unecritinib, and gastrointestinal or neurological events with other agents.

#### 3.4 Heterogeneity assessment

Significant heterogeneity was anticipated in the proportional meta-analysis, primarily due to variations in demographic and clinical characteristics of study populations, as well as geographical disparities across studies (Migliavaca et al., 2022). Among the ROS1-TKIs in this study, crizotinib and ceritinib were included in a relatively higher number of studies, while other agents were limited to 3 or fewer studies. Notably, ceritinib demonstrated low heterogeneity in both all-grade AEs and SAEs across meta-analyses. Consequently, subgroup analyses to explore heterogeneity were conducted exclusively for crizotinib, which exhibited substantial heterogeneity in the pooled incidence of AEs (I^2^ = 68.88%) and SAEs (I^2^ = 88.76%).

Subgroup analyses were stratified by gender distribution (≤55% vs. >55% female), ethnicity (Asian vs. multiethnic cohorts), publication period (before 2018 vs. after 2018), study design (RCTs vs. single-arm trials), and age (≤54 vs. >54 years). As shown in Supplementary Figures S3–S4 and summarized in Supplementary Table S4, significant heterogeneity in all-grade AEs associated with crizotinib was observed across ethnicity subgroups (*P* = 0.003), while heterogeneity in SAEs was linked to study design (*P* < 0.001). No other subgroups significantly influenced heterogeneity for AEs or SAEs. The incidence of crizotinib related AEs in the Asian population was lower than that in the pan-racial population, while the incidence of crizotinib related SAEs in RCTs was lower than that in single-arm studies. These findings suggest that the risk of crizotinib-related AEs may be lower in Asian populations compared to other ethnic groups, and that single-arm designs may be associated with higher AE rates. Additionally, the limited number of included studies may further amplify variability in pooled estimates.

#### 3.5 Sensitivity assessment

**FIGURE 3:**
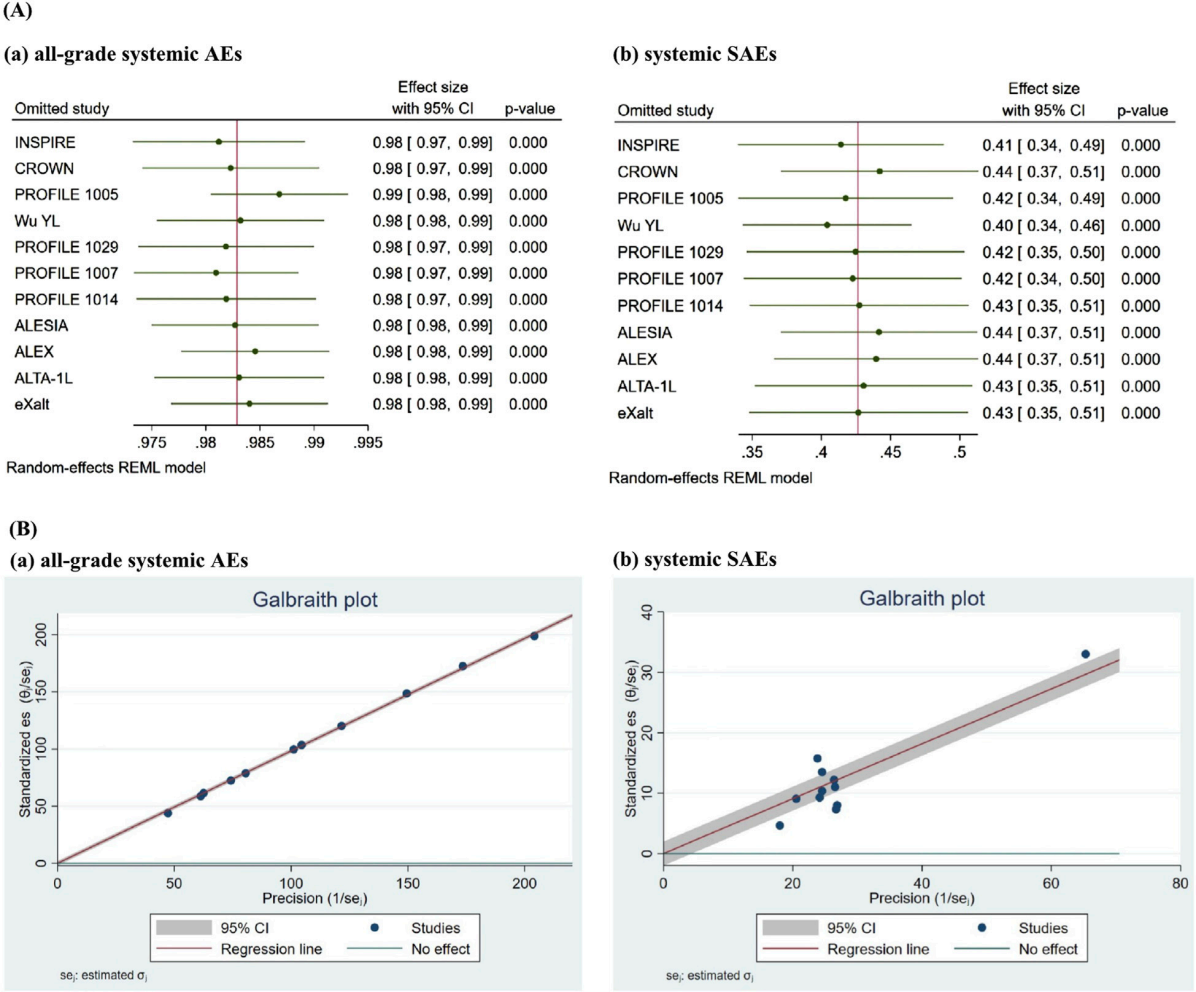
Sensitivity analysis of crizotinib-related AEs and SAEs was performed using leave-one-out analysis and Galbraith plot. The leave-one-out sensitivity analysis **(A)** demonstrated that sequential omission of individual studies did not substantially alter the pooled incidence estimates for all-grade AEs or SAEs associated with crizotinib treatment. The Galbraith plot **(B)** revealed that most study points lie within the 95% CI (gray shaded area) and cluster relatively closely around the regression line (red line), with only a few points showing minor deviations. No obvious indication of publication bias was observed.

Considering the number of studies for each ROS1-TKI, sensitivity asssessment were conducted for crizotinib. The leave-one-out sensitivity analysis (Figure 3A) demonstrated that sequential omission of individual studies did not substantially alter the pooled incidence estimates for all-grade AEs (0.98, 95% CI 0.97–0.99) or SAEs (0.43, 95% CI 0.36–0.49) associated with crizotinib treatment. As previously indicated, study design was identified as a potential source of heterogeneity in SAE incidence associated with crizotinib. To further investigate this, we conducted a subgroup analysis restricted to RCTs, excluding two single-arm studies (Supplementary Figure S5). The refined analysis revealed comparable AE incidence (0.99, 95% CI 0.97–1.00) but a modest reduction in SAE incidence (0.39, 95% CI 0.33–0.45). This represents an absolute risk reduction of 4% compared to the overall SAE estimate derived from all studies (0.43, 95% CI 0.36–0.49), while AE rates remained stable between analyses. The Galbraith plot showed that no obvious publication bias was observed in studies reporting the incidence of crizotinib-related AEs or SAEs (Figure 3B). This observation suggests the need for cautious interpretation of SAE estimates, particularly regarding potential underreporting in non-randomized studies.

## 4 Discussion

Toxicity profiles of small-molecule target therapy drugs in NSCLC patients hold significant clinical implications for optimizing therapeutic strategies and patient safety. This proportional meta-analysis presents the first comprehensive evaluation of ROS1-TKIs’ toxicity profiles in NSCLC patients. According to the pooled incidence of systemic SAEs, repotrectinib might be more tolerable, while unecitinib might have a lower safety profile. Specific AEs exhibited heterogeneous safety profiles: repotrectinib was associated with a higher incidence of dizziness, while entrectinib showed a tendency toward frequent fatigue, and lorlatinib exhibited an increased rate of edema. Notably, both taletrectinib and unecritinib were linked to hepatotoxicity. Gastrointestinal adverse events emerged as a predominant issue across ceritinib, taletrectinib, crizotinib, and unecritinib. Additionally, taletrectinib was characterized by a higher occurrence of anemia; unecritinib was associated with elevated rates of neutropenia and sinus bradycardia; and crizotinib showed a greater incidence of ocular disorders. These findings present the first comprehensive evaluation of ROS1-TKIs toxicity profiles in NSCLC patients, offering critical insights to guide drug selection and underscoring the necessity of rigorous safety monitoring throughout the treatment course.

In previous studies, the toxicity profiles of TKIs in lung cancer therapy have been extensively studied for EGFR and ALK inhibitors. Meta-analyses have been performed among EGFR-TKIs (Zhao et al., 2021) and ALK inhibitors (Luo et al., 2023), respectively. Recently, several small-scale meta-analysis studies were published to compare the safety of a few EGFR-TKIs (Osimertinib, Erlotinib, and Gefitinib) (Qureshi et al., 2025) or individual adverse reactions (diarrhea, infection, rash) (Lai et al., 2024). In the realm of ALK inhibitors, non-comparative assessments through meta-analysis were carried out for pairs of drugs, such as crizotinib vs. Alectinib (Xiong et al., 2023) and Alectinib vs. lorlatinib (Attili et al., 2024). However, systematic reviews or meta-analyses on inhibitors targeting ROS1, KRAS, MET, etc., remain less characterized. Our study represents the largest and most comprehensive analysis to date evaluating the toxicity profiles of ROS1-TKIs in patients with NSCLC using proportional meta-analysis.

Proportional meta-analysis was chosen over network meta-analysis in this study due to its methodological advantages. Firstly, proportional meta-analysis overcomes reliance on direct comparisons. Conventional meta-analysis fails without head-to-head data, while network meta-analysis requires a connected trial network and untestable transitivity assumptions, which are often invalid in sparse datasets. Proportional meta-analysis aggregates absolute event rates from single-arm studies or one of the intervention groups of RCTs, enabling robust treatment-specific estimates even with no direct or indirect comparisons. Secondly, it is suitable for rare diseases or emerging therapies. For conditions like ROS1-positive NSCLC in our study, head-to-head RCTs are logistically challenging, leaving single-arm or small studies as primary data source. Proportional meta-analysis generates stable pooled outcomes (e.g., toxicity rates) for these studies. An increasing number of studies on targeted therapies are adopting single-arm designs, and single-arm rate meta-analysis will thus play an important role in the future.

By integrating data from multiple clinical trials, both RCTs and single-arm studies, we systematically assessed both broad-spectrum AEs and specific toxicities. These safety profiles enable personalized therapy tailored to patients’ health status and risk factors, enhance proactive side effect management to improve adherence and outcomes, support informed drug selection that balances efficacy and tolerability, and inform targeted monitoring for early intervention.

Due to the limited number of eligible studies, heterogeneity assessments in this analysis were confined exclusively to crizotinib-associated AEs and SAEs. Substantial heterogeneity in crizotinib-related AE/SAE incidences was linked to ethnicity and study design, corroborating that genetic polymorphisms and unblinded trial designs may influence AE reporting and susceptibility. The open-label nature of all included RCTs likely introduced performance bias, potentially inflating AE rates due to heightened surveillance [8]. Sensitivity analyses confirmed the robustness of crizotinib’s pooled estimates, but the limited number of studies for newer agents (e.g., iruplinalkib, unecritinib) precluded similar assessments, warranting cautious interpretation.

This study has several limitations. In terms of methodology, to date, there are very few head to head RCT studies on ROS1 inhibitors, and direct comparative toxicity evaluations between agents cannot be obtained. While the small number of included studies for certain inhibitors (≤3 studies per ROS1-TKI, with some TKIs only having one study) may reduce the stability and precision of incidence estimates. We will continue to closely monitor RCT research in this field and strive to further refine our work in the future. In terms of generalizability, the findings is constrained by the exclusion criteria of clinical trials, which systematically omit vulnerable subgroups such as elderly patients and those with hepatic/renal impairment, highlighting the need for real-world studies to complement these findings.

## 5 Conclusion

This proportional meta-analysis elucidates the safety profiles of ROS1-TKIs, comprehensively covering the overall incidences, spectrum, and severity of AEs. All the investigated agents demonstrate notably high rates of all-grade AEs. Given the distinct patterns of SAEs and subtype-specific toxicities among these agents, individualized management approaches are imperative. Clinicians should carefully balance these safety profiles against efficacy data and patient’s comorbid conditions to achieve optimal therapeutic outcomes. Real-world studies will be necessary to conduct in the future to characterize the toxicity profiles of ROS1-TKIs.

## Data Availability

The original contributions presented in the study are included in the article/Supplementary Material, further inquiries can be directed to the corresponding author.

## References

[B1] Attili I. Fuorivia V. Spitaleri G. Corvaja C. Trillo Aliaga P. Del Signore E. (2024). Alectinib vs. Lorlatinib in the front-line setting for ALK-rearranged non-small-cell lung cancer (NSCLC): a deep dive into the main differences across ALEX and CROWN phase 3 trials. Cancers 16 (13), 2457. 10.3390/cancers16132457 39001519 PMC11240527

[B2] Blackhall F. Ross Camidge D. Shaw A. T. Soria J.-C. Solomon B. J. Mok T. (2017). Final results of the large-scale multinational trial PROFILE 1005: efficacy and safety of crizotinib in previously treated patients with advanced/metastatic ALK-positive non-small-cell lung cancer. ESMO Open 2 (3), e000219. 10.1136/esmoopen-2017-000219 29209525 PMC5703388

[B3] Camidge D. R. Kim H. R. Ahn M.-J. Yang J. C. H. Han J.-Y. Hochmair M. J. (2020). Brigatinib versus crizotinib in advanced ALK inhibitor–naive ALK-positive non–small cell lung cancer: second interim analysis of the phase III ALTA-1L trial. J. Clin. Oncol. 38 (31), 3592–3603. 10.1200/jco.20.00505 32780660 PMC7605398

[B5] Crinò L. Ahn M.-J. De Marinis F. Groen H. J. M. Wakelee H. Hida T. (2016). Multicenter phase II study of whole-body and intracranial activity with ceritinib in patients with ALK-rearranged non–small-cell lung cancer previously treated with chemotherapy and crizotinib: results from ASCEND-2. J. Clin. Oncol. 34 (24), 2866–2873. 10.1200/jco.2015.65.5936 27432917

[B6] Davies K. D. Doebele R. C. (2013). Molecular Pathways: ROS1 fusion proteins in cancer. Clin. Cancer Res. 19 (15), 4040–4045. 10.1158/1078-0432.Ccr-12-2851 23719267 PMC3732549

[B7] Drilon A. Siena S. Dziadziuszko R. Barlesi F. Krebs M. G. Shaw A. T. (2020). Entrectinib in ROS1 fusion-positive non-small-cell lung cancer: integrated analysis of three phase 1–2 trials. Lancet Oncol. 21 (2), 261–270. 10.1016/s1470-2045(19)30690-4 31838015 PMC7811790

[B8] Drilon A. Camidge D. R. Lin J. J. Kim S. W. Solomon B. J. Dziadziuszko R. (2024). Repotrectinib in *ROS1* fusion-positive non-small-cell lung cancer. N. Engl. J. Med. 390 (2), 118–131. 10.1056/NEJMoa2302299 38197815 PMC11702311

[B9] Dziadziuszko R. Krebs M. G. De Braud F. Siena S. Drilon A. Doebele R. C. (2021a). Updated integrated analysis of the efficacy and safety of entrectinib in locally advanced or MetastaticROS1Fusion–positive non–small-cell lung cancer. J. Clin. Oncol. 39 (11), 1253–1263. 10.1200/jco.20.03025 33646820 PMC8078299

[B10] Dziadziuszko R. Krebs M. G. De Braud F. Siena S. Drilon A. Doebele R. C. (2021b). Updated integrated analysis of the efficacy and safety of entrectinib in locally advanced or metastatic ROS1 fusion-positive non-small-cell lung cancer. J. Clin. Oncol. 39 (11), 1253–1263. 10.1200/JCO.20.03025 33646820 PMC8078299

[B11] Ferlay J. E. M. Lam F. Laversanne M. Colombet M. Mery L. Piñeros M. (2024). Global cancer observatory: cancer today. Lyon, France. Lyon: International Agency for Research on Cancer. Available online at: https://gco.iarc.who.int/today (Accessed December 11, 2024).

[B12] Ganti A. K. Klein A. B. Cotarla I. Seal B. Chou E. (2021). Update of incidence, prevalence, survival, and initial treatment in patients with non-small cell lung cancer in the US. JAMA Oncol. 7 (12), 1824–1832. 10.1001/jamaoncol.2021.4932 34673888 PMC8532041

[B13] Gendarme S. Bylicki O. Chouaid C. Guisier F. (2022). ROS-1 fusions in non-small-cell lung cancer: evidence to date. Curr. Oncol. 29 (2), 641–658. 10.3390/curroncol29020057 35200557 PMC8870726

[B14] Hida T. Seto T. Horinouchi H. Maemondo M. Takeda M. Hotta K. (2018). Phase II study of ceritinib in alectinib‐pretreated patients with anaplastic lymphoma kinase‐rearranged metastatic non‐small‐cell lung cancer in Japan: ASCEND‐9. Cancer Sci. 109 (9), 2863–2872. 10.1111/cas.13721 29959809 PMC6125456

[B15] Higgins JP T. S. Deeks J. J. Altman D. G. (2003). Measuring inconsistency in meta-analyses. BMJ 327 (7414), 557–560. 10.1136/bmj.327.7414.557 12958120 PMC192859

[B16] Horn L. Wang Z. Wu G. Poddubskaya E. Mok T. Reck M. (2021). Ensartinib vs crizotinib for patients with anaplastic lymphoma kinase-positive non-small cell lung cancer: a randomized clinical trial. JAMA Oncol. 7 (11), 1617–1625. 10.1001/jamaoncol.2021.3523 34473194 PMC8414368

[B17] Lai X. Zeng J. Xiao Z. Xiao J. (2024). Efficacy and safety of EGFR-TKIs for non-small cell lung cancer: a meta-analysis of randomized controlled clinical trials. Medicine 103 (23), e38277. 10.1097/md.0000000000038277 38847673 PMC11155537

[B19] Li W. Xiong A. Yang N. Fan H. Yu Q. Zhao Y. (2024). Efficacy and safety of taletrectinib in Chinese patients with ROS1+ non–small cell lung cancer: the phase II TRUST-I study. J. Clin. Oncol. 42 (22), 2660–2670. 10.1200/jco.24.00731 38822758 PMC11272140

[B20] Lim S. M. Kim H. R. Lee J.-S. Lee K. H. Lee Y.-G. Min Y. J. (2017). Open-label, multicenter, phase II study of ceritinib in patients with non–small-cell lung cancer harboring ROS1 rearrangement. J. Clin. Oncol. 35 (23), 2613–2618. 10.1200/jco.2016.71.3701 28520527

[B21] Lu S. Zhou Q. Liu X. Du Y. Fan Y. Cheng Y. (2022). Lorlatinib for previously treated ALK-positive advanced NSCLC: primary efficacy and safety from a phase 2 study in People’s Republic of China. J. Thorac. Oncol. 17 (6), 816–826. 10.1016/j.jtho.2022.02.014 35307611

[B22] Lu S. Pan H. Wu L. Yao Y. He J. Wang Y. (2023). Efficacy, safety and pharmacokinetics of Unecritinib (TQ-B3101) for patients with ROS1 positive advanced non-small cell lung cancer: a Phase I/II Trial. Signal Transduct. Target. Ther. 8 (1), 249. 10.1038/s41392-023-01454-z 37385995 PMC10310851

[B23] Luo Y. Zhang Z. Guo X. Tang X. Li S. Gong G. (2023). Comparative safety of anaplastic lymphoma kinase tyrosine kinase inhibitors in advanced anaplastic lymphoma kinase-mutated non-small cell lung cancer: systematic review and network meta-analysis. Lung Cancer 184, 107319. 10.1016/j.lungcan.2023.107319 37597303

[B24] Migliavaca C. B. Stein C. Colpani V. Barker T. H. Ziegelmann P. K. Munn Z. (2022). Meta‐analysis of prevalence: I2 statistic and how to deal with heterogeneity. Res. Synthesis Methods 13 (3), 363–367. 10.1002/jrsm.1547 35088937

[B25] Network N. C. C. (2024). Clinical practice guidelines in oncology for non-small cell lung cancer. Version 11. Available online at: https://www.nccn.org/guidelines/guidelines-detail?category=1&id=1450 (Accessed December 16, 2024).

[B26] Nishio M. Felip E. Orlov S. Park K. Yu C.-J. Tsai C.-M. (2020). Final overall survival and other efficacy and safety results from ASCEND-3: phase II study of ceritinib in ALKi-naive patients with ALK-rearranged NSCLC. J. Thorac. Oncol. 15 (4), 609–617. 10.1016/j.jtho.2019.11.006 31778798

[B150] Pharmaceuticals N. (2019). LDK378 in adult Chinese patients with ALK-rearranged (ALK-positive) advanced non-small cell lung cancer (NSCLC) previously treated with crizotinib. [Online]. Available online at: https://clinicaltrials.gov/study/NCT02040870?intr=ceritinib&cond=NCT02040870&rank=1 (Accessed April 3, 2025).

[B27] Peters S. Camidge D. R. Shaw A. T. Gadgeel S. Ahn J. S. Kim D.-W. (2017). Alectinib versus crizotinib in untreated ALK-positive non–small-cell lung cancer. N. Engl. J. Med. 377 (9), 829–838. 10.1056/NEJMoa1704795 28586279

[B151] Pfizer (2017). An investigational drug, PF-02341066 is being studied versus standard of care in patients with advanced non-small cell lung cancer with a specific gene profile involving the anaplastic lymphoma kinase (ALK) gene. *[Online]* . Available online at: https://clinicaltrials.gov/study/NCT00932893?intr=ceritinib&cond=NCT00932893&rank=1 (Accessed February 1, 2025).

[B28] Qureshi Z. Altaf F. Jamil A. Siddique R. (2025). Meta-analysis of targeted therapies in EGFR-mutated non-small cell lung cancer: efficacy and safety of osimertinib, Erlotinib, and Gefitinib as first-line treatment. Am. J. Clin. Oncol. 48 (1), 44–54. 10.1097/coc.0000000000001138 39257317

[B29] Seto T. Hayashi H. Satouchi M. Goto Y. Niho S. Nogami N. (2020). Lorlatinib in previously treated anaplastic lymphoma kinase‐rearranged non–small cell lung cancer: Japanese subgroup analysis of a global study. Cancer Sci. 111 (10), 3726–3738. 10.1111/cas.14576 32681682 PMC7540988

[B30] Shaw A. T. Ou S.-H. I. Bang Y.-J. Camidge D. R. Solomon B. J. Salgia R. (2014). Crizotinib in ROS1-rearranged non–small-cell lung cancer. N. Engl. J. Med. 371 (21), 1963–1971. 10.1056/NEJMoa1406766 25264305 PMC4264527

[B31] Shaw A. T. Kim T. M. Crino L. Gridelli C. Kiura K. Liu G. (2017). Ceritinib versus chemotherapy in patients with *ALK*-rearranged non-small-cell lung cancer previously given chemotherapy and crizotinib (ASCEND-5): a randomised, controlled, open-label, phase 3 trial. Lancet Oncol. 18 (7), 874–886. 10.1016/s1470-2045(17)30339-x 28602779

[B32] Shaw A. T. Bauer T. M. de Marinis F. Felip E. Goto Y. Liu G. (2020). First-line lorlatinib or crizotinib in advanced *ALK*-positive lung cancer. N. Engl. J. Med. 383 (21), 2018–2029. 10.1056/NEJMoa2027187 33207094

[B33] Shi Y. Fang J. Hao X. Zhang S. Liu Y. Wang L. (2022). Safety and activity of WX-0593 (Iruplinalkib) in patients with ALK- or ROS1-rearranged advanced non-small cell lung cancer: a phase 1 dose-escalation and dose-expansion trial. Signal Transduct. Target Ther. 7 (1), 25. 10.1038/s41392-021-00841-8 35087031 PMC8795197

[B34] Shi Y. Chen J. Zhang H. Zhang Z. Zhang Y. Wang Z. (2023). Efficacy and safety of iruplinalkib (WX-0593) in ALK-positive crizotinib-resistant advanced non-small cell lung cancer patients: a single-arm, multicenter phase II study (INTELLECT). BMC Med. 21 (1), 72. 10.1186/s12916-023-02738-5 36829154 PMC9960473

[B35] Shi Y. Chen J. Yang R. Wu H. Wang Z. Yang W. (2024). Iruplinalkib (WX-0593) versus crizotinib in ALK TKI-naive locally advanced or metastatic ALK-positive NSCLC: interim analysis of a randomized, open-label, phase 3 study (INSPIRE). J. Thorac. Oncol. 19 (6), 912–927. 10.1016/j.jtho.2024.01.013 38280448

[B36] Solomon B. J. Besse B. Bauer T. M. Felip E. Soo R. A. Camidge D. R. (2018a). Lorlatinib in patients with ALK-positive non-small-cell lung cancer: results from a global phase 2 study. Lancet Oncol. 19 (12), 1654–1667. 10.1016/s1470-2045(18)30649-1 30413378

[B37] Solomon B. J. Kim D. W. Wu Y. L. Nakagawa K. Mekhail T. Felip E. (2018b). Final overall survival analysis from a study comparing first-line crizotinib versus chemotherapy in ALK-Mutation-positive non-small-cell lung cancer. J. Clin. Oncol. 36 (22), 2251–2258. 10.1200/jco.2017.77.4794 29768118

[B38] Soria J.-C. Tan D. S. W. Chiari R. Wu Y.-L. Paz-Ares L. Wolf J. (2017a). First-line ceritinib versus platinum-based chemotherapy in advanced ALK -rearranged non-small-cell lung cancer (ASCEND-4): a randomised, open-label, phase 3 study. Lancet 389 (10072), 917–929. 10.1016/s0140-6736(17)30123-x 28126333

[B39] Soria J. C. Tan D. S. W. Chiari R. Wu Y. L. Paz-Ares L. Wolf J. (2017b). First-line ceritinib versus platinum-based chemotherapy in advanced ALK-rearranged non-small-cell lung cancer (ASCEND-4): a randomised, open-label, phase 3 study. Lancet 389 (10072), 917–929. 10.1016/S0140-6736(17)30123-X 28126333

[B40] Sterne J. A. C. Savović J. Page M. J. Elbers R. G. Blencowe N. S. Boutron I. (2019). RoB 2: a revised tool for assessing risk of bias in randomised trials. Bmj 366, l4898. 10.1136/bmj.l4898 31462531

[B41] Wu Y. L. Lu S. Lu Y. Zhou J. Y. Shi Y. K. Sriuranpong V. (2018). Results of PROFILE 1029, a phase III comparison of first-line crizotinib versus chemotherapy in east asian patients with *ALK*-positive advanced non-small cell lung cancer. J. Thorac. Oncol. 13 (10), 1539–1548. 10.1016/j.jtho.2018.06.012 29966800

[B42] Wu Y.-L. Lu S. Yang J.C.-H. Zhou J. Seto T. Ahn M.-J. (2022). Final overall survival, safety, and quality of life results from a phase 2 study of crizotinib in east asian patients with ROS1-positive advanced NSCLC. JTO Clin. Res. Rep. 3 (10), 100406. 10.1016/j.jtocrr.2022.100406 36247019 PMC9558051

[B43] Xiong R. Fu H. Zhang Q. Li W. (2023). Safety and efficacy of alectinib versus crizotinib in alk-positive non-small cell lung cancer: an update meta-analysis. Pak J. Pharm. Sci. 36 (2), 365–372. 37530142

[B44] Zhao Y. Cheng B. Chen Z. Li J. Liang H. Chen Y. (2021). Toxicity profile of epidermal growth factor receptor tyrosine kinase inhibitors for patients with lung cancer: a systematic review and network meta-analysis. Crit. Rev. Oncology/Hematology 160, 103305. 10.1016/j.critrevonc.2021.103305 33757838

[B45] Zhou C. Kim S.-W. Reungwetwattana T. Zhou J. Zhang Y. He J. (2019). Alectinib versus crizotinib in untreated Asian patients with anaplastic lymphoma kinase-positive non-small-cell lung cancer (ALESIA): a randomised phase 3 study. Lancet Respir. Med. 7 (5), 437–446. 10.1016/s2213-2600(19)30053-0 30981696

