# Supplementary material for: Toxicity profiles of ROS1 tyrosine kinase inhibitors in advanced non-small cell lung cancer: a systematic review and proportional meta-analysis
Source: Front Pharmacol. 2025 Aug 29;16:1644034. doi: 10.3389/fphar.2025.1644034 (PMC12426102; doi:10.3389/fphar.2025.1644034)
Supplement: Supplementary file 1 [file Supplementaryfile1.docx]

**Toxicity profiles of ROS 1 tyrosine kinase inhibitors in non-small cell lung cancer: a proportional meta-analysis**

Supplementary materials

**Table S1.** Literature search strategy.

**Table S2.** Excluded studies with reasons.

**Table S3.** The risk of bias assessments of single-arm studies via ROBINS-I V2 tool.

**Table S4.** The subgroup estimates for gender, ethnicity, age, study type, publication date.

**Figure S1.** Flow diagram for the selection of eligible studies.

**Figure S2.** The risk of bias assessments of RCTs via Cochrane Risk of Bias Tool.

**Figure S3.** Forest plots of subgroup analysis of heterogeneity in the incidence of systemic all-grade AEs associated with Crizotinib.

**Figure S4.** Forest plots of subgroup analysis of heterogeneity in the incidence of systemic SAEs associated with Crizotinib.

**Figure S5.** Forest plots of incidences of all-grade AEs (A) and SAEs (B) restricting RCTs.

Table S1. Literature search strategy.

**Pubmed**

| #1 | (((((((((((NSCLC[Title/Abstract]) OR (Non-Small Cell Lung Cancer[Title/Abstract])) OR (Carcinoma, Non Small Cell Lung[Title/Abstract])) OR (Carcinomas, Non-Small-Cell Lung[Title/Abstract])) OR (Lung Carcinoma, Non-Small-Cell[Title/Abstract])) OR (Lung Carcinomas, Non-Small-Cell[Title/Abstract])) OR (Non-Small-Cell Lung Carcinomas[Title/Abstract])) OR (Carcinoma, Non-Small Cell Lung[Title/Abstract])) OR (Non-Small-Cell Lung Carcinoma[Title/Abstract])) OR (Non Small Cell Lung Carcinoma[Title/Abstract])) OR (Nonsmall Cell Lung Cancer[Title/Abstract])) OR (Non-Small Cell Lung Carcinoma[Title/Abstract]) |
| --- | --- |
| #2 | (((((((((((((((((((((Crizotinib[Title/Abstract])) OR (Entrectinib[Title/Abstract])) OR (Lorlatinib[Title/Abstract])) OR (Ceritinib[Title/Abstract])) OR (Repotrectinib[Title/Abstract])) OR (Unecritinib[Title/Abstract])) OR (Iruplinalkib[Title/Abstract])) OR (Taletrectinib[Title/Abstract])) OR (PF-02341066[Title/Abstract])) OR (PF 02341066[Title/Abstract])) OR (PF02341066[Title/Abstract])) OR (PF-2341066[Title/Abstract])) OR (PF2341066[Title/Abstract])) OR (PF 2341066[Title/Abstract])) OR (Xalkori[Title/Abstract])) OR (Zykadia[Title/Abstract])) OR (LDK378[Title/Abstract])) OR (PF-06463922[Title/Abstract])) OR (PF06463922[Title/Abstract])) OR (Lorbrena[Title/Abstract])) OR (Rozlytrek[Title/Abstract]) OR (RXDX-101[Title/Abstract]) OR (NMS-E628[Title/Abstract]) OR (DS6051b[Title/Abstract]) OR (AB-106[Title/Abstract]) |
| #3 | (((((((Randomized Controlled Trial[Publication Type]) OR (Clinical Trial[Publication Type])) OR (trial[Title/Abstract])) OR (randomized[Title/Abstract])) OR (randomised[Title/Abstract])) OR (randomly[Title/Abstract])) OR (phase[Title/Abstract])) OR (single-arm[Title/Abstract]) OR (single arm[Title/Abstract]) |
| #4 | "2013/01/01"[Date - Publication] : "2025/2/28"[Date - Publication] |
| #5 | #1 AND #2 AND #3 AND #4 AND #5 |

**Embase**

| #1 | nsclc OR 'non-small cell lung cancer':ab,ti OR 'carcinoma, non small cell lung':ab,ti OR 'carcinomas, non-small-cell lung':ab,ti OR 'lung carcinoma, non-small-cell':ab,ti OR 'lung carcinomas, non-small-cell':ab,ti OR 'non-small-cell lung carcinomas':ab,ti OR 'carcinoma, non-small cell lung':ab,ti OR 'non-small-cell lung carcinoma':ab,ti OR 'non small cell lung carcinoma':ab,ti OR 'nonsmall cell lung cancer':ab,ti OR 'non-small cell lung carcinoma':ab,ti |
| --- | --- |
| #2 | 'tyrosine kinase inhibitor'/exp OR 'tyrosine kinase inhibitor' OR (('tyrosine'/exp OR tyrosine) AND ('kinase'/exp OR kinase) AND ('inhibitor'/exp OR inhibitor)) OR 'kinase inhibitor':ab,ti OR crizotinib:ab,ti OR entrectinib:ab,ti OR lorlatinib:ab,ti OR ceritinib:ab,ti OR repotrectinib:ab,ti OR unecritinib:ab,ti OR iruplinalkib:ab,ti OR taletrectinib:ab,ti OR 'pf 02341066':ab,ti OR pf02341066:ab,ti OR pf2341066:ab,ti OR 'pf 2341066':ab,ti OR xalkori:ab,ti OR zykadia:ab,ti OR ldk378:ab,ti OR 'pf 06463922':ab,ti OR pf06463922:ab,ti OR lorbrena:ab,ti OR rozlytrek:ab,ti OR 'rxdx 101':ab,ti OR 'nms e628':ab,ti |
| #3 | [controlled clinical trial]/lim OR [randomized controlled trial]/lim OR [single-arm trial]/lim |
| #4 | #1 AND #2 AND #3 AND #4 |

**Cochrane Library**

| #1 | “NSCLC” OR“Non-Small Cell Lung Cancer” OR“Carcinomas, Non-Small-Cell Lung” OR“Lung Carcinoma, Non-Small-Cell” OR“Lung Carcinomas, Non-Small-Cell” OR“Non-Small-Cell Lung Carcinomas” OR“Carcinoma, Non-Small Cell Lung” OR“Non-Small-Cell Lung Carcinoma” OR“Non Small Cell Lung Carcinoma” OR“Nonsmall Cell Lung Cancer” OR“Non-Small Cell Lung Carcinoma” in Title Abstract Keyword (Word variations have been searched) |
| --- | --- |
| #2 | “Tyrosine Kinase Inhibitor” OR “Kinase Inhibitor” OR TKI OR Crizotinib OR Entrectinib OR Lorlatinib OR Ceritinib OR Unecritinib OR Iruplinalkib OR Taletrectinib OR PF-02341066 OR PF 02341066 OR PF02341066 OR PF-2341066 OR PF2341066 OR PF 2341066 OR Xalkori OR Zykadia OR LDK378 OR PF-06463922 OR PF06463922 OR Lorbrena OR Rozlytrek OR RXDX-101 OR Repotrectinib OR NMS-E628 in TitleAbstract Keyword |
| #3 | "randomized controlled trial" OR "randomized-controlled trials" OR "single-arm trial" in Publication Type OR "trial " OR "randomized " OR "randomised " OR "randomly" OR "phase" in Title Abstract Keyword |
| #4 | #1 AND #2 AND #3 AND #4 |

**ClinicalTrials.gov**

| #1 | (“lung cancer” OR “lung carcinoma”) AND (“ROS proto-oncogene 1” OR ROS1) AND ( Crizotinib OR Entrectinib OR Lorlatinib OR Ceritinib OR Entrectinib OR Unecritinib OR Iruplinalkib OR Taletrectinib OR PF-02341066 OR PF 02341066 OR PF02341066 OR PF-2341066 OR PF2341066 OR PF 2341066 OR Xalkori OR Zykadia OR LDK378 OR PF-06463922 OR PF06463922 OR Lorbrena OR Rozlytrek OR RXDX-101 OR NMS-E628 OR DS6051b OR AB-106) AND (randomized OR randomized OR randomly OR random OR phase OR single) |
| --- | --- |

Table **S2**. Excluded studies with reasons.

| **Author,year/NCT number** | **Title** | **Drugs** | **Reason for exclusion** |
| --- | --- | --- | --- |
| ***D.Moro-Sibilot, 2019*** | **Crizotinib in c-MET- or ROS1-positive NSCLC: results of the AcSe´ phase II trial** | **Crizotinib** | Not reported detailed safety data |
| ***Robert C Doebele*** | **Comparative effectiveness analysis between entrectinib clinical trial and crizotinib real-world data in *ROS1*+ NSCLC** | **Entrectinib+ Crizotinib** | Not reported detailed safety data |
| ***Fredrik O. L. Nilsson,2021*** | **The Cost‑Efectiveness of Lorlatinib Versus Chemotherapy as a Second‑ or Third‑Line Treatment in Anaplastic Lymphoma Kinase (*ALK*)‑Positive Non‑small‑cell Lung Cancer in Sweden** | **Lorlatinib+** C**hemotherapy** | Not reported detailed safety data |
| ***Yicong Bian,2024*** | **Pharmacokinetics, metabolism, excretion and safety of iruplinalkib (WX-0593), a novel ALK inhibitor, in healthy subjects: a phase I human radiolabeled mass balance study** | **Iruplinalkib** | Not reported detailed safety data |
| ***Nagasaka M,2023*** | **TRUST-II: a global phase II study of taletrectinib in ROS1-positive non-small-celllung cancer and other solid tumors** | **Taletrectinib** | Not reported detailed safety data |
| ***Solange Peters,2024*** | **Entrectinib in ROS1-positive advanced non-small cell lung cancer: the phase 2/3 BFAST trial** | **Entrectinib** | Not reported detailed safety data |
| ***Joseph Chen, 2021*** | **Lorlatinib Exposure-Response Analyses for Safety and Efficacy in a Phase I/II Trial to Support Benefit–Risk Assessment in Non-Small Cell Lung Cancer** | **Lorlatinib** | Not reported detailed safety data |
| ***Drilon, A，2017*** | **Safety and Antitumor Activity of the Multitargeted Pan-TRK, ROS1, and ALK Inhibitor Entrectinib: Combined Results from Two Phase I Trials (ALKA-372-001 and STARTRK-1)** | **Entrectinib** | Phase I |
| ***Shaw, A. T，2019*** | **Crizotinib in ROS1-rearranged advanced non-small-cell lung cancer (NSCLC): updated results, including overall survival, from PROFILE 1001** | **Crizotinib** | Phase I |
| ***Shi, Y，2022*** | **Safety and activity of WX-0593 (Iruplinalkib) in patients with ALK- or ROS1-rearranged advanced non-small cell lung cancer: a phase 1 dose-escalation and dose-expansion trial** | **Iruplinalkib** | Phase I |
| ***Pasi A., 2016*** | **Combined Pan-HER and ALK/ROS1/MET Inhibition with Dacomitinib and Crizotinib in Advanced Non–Small Cell Lung Cancer: Results of a Phase I Study** | **Dacomitinib+Crizotinib** | Other ALK |
| ***NCT02838420*** | **A Study to Evaluate and Compare the Efficacy and Safety of Alectinib Versus Crizotinib and to Evaluate the Pharmacokinetics of Alectinib in Asian Participants With Treatment-Naive Anaplastic Lymphoma Kinase (ALK)-Positive Advanced Non-Small Cell Lung Cancer (NSCLC)** | **Crizotinib vs Alectinib** | Other ALK |
| ***NCT02767804*** | **eXalt3: Study Comparing X-396 (Ensartinib) to Crizotinib in ALK Positive Non-Small Cell Lung Cancer (NSCLC) Patients** | **Crizotinib vs Ensartinib** | Other ALK |
| ***NCT02737501*** | **ALTA-1L Study: A Study of Brigatinib Versus Crizotinib in Anaplastic Lymphoma Kinase Positive (ALK+) Advanced Non-small Cell Lung Cancer (NSCLC) Participants** | **Crizotinib vs Brigatinib** | Other ALK |
| ***NCT02075840*** | **A Study Comparing Alectinib With Crizotinib in Treatment-Naive Anaplastic Lymphoma Kinase-Positive Advanced Non-Small Cell Lung Cancer Participants** | **Crizotinib vs Alectinib** | Other ALK |
| ***Shaw AT, 2013*** | **Crizotinib versus chemotherapy in advanced ALK-positive lung cancer** | **Crizotinib vs** C**hemotherapy** | Overlap with Blackhall F, 2014 |
| ***Nishio M, 2018*** | **Crizotinib versus Chemotherapy in Asian Patients with ALK-Positive Advanced Non-small Cell Lung Cancer** | **Crizotinib vs** C**hemotherapy** | Overlap with Blackhall F, 2014 |
| ***Solomon BJ, 2014*** | **First-line crizotinib versus chemotherapy in ALK-positive lung cancer** | **Crizotinib vs** C**hemotherapy** | Overlap with Solomon BJ, 2018 |
| ***Solomon BJ, 2016*** | **Intracranial Efficacy of Crizotinib Versus Chemotherapy in Patients With Advanced ALK-Positive Non-Small-Cell Lung Cancer: Results From PROFILE 1014** | **Crizotinib vs** C**hemotherapy** | Overlap with Solomon BJ, 2018 |
| ***Wilner KD, 2019*** | **Comparison of cardiovascular effects of crizotinib and chemotherapy in ALK-positive advanced non-small-cell lung cancer** | **Crizotinib vs** C**hemotherapy** | Overlap with Solomon BJ, 2018 |
| ***Li J, 2019*** | **Comparative efficacy of first-line ceritinib and crizotinib in advanced or metastatic anaplastic lymphoma kinase-positive non-small cell lung cancer: an adjusted indirect comparison with external controls** | **Ceritinib vs Chemotherapy** | Overlap with Soria JC, 2017 |
| ***Solomon BJ, 2023*** | **Efficacy and safety of first-line lorlatinib versus crizotinib in patients with advanced, ALK-positive non-small-cell lung cancer: updated analysis of data from the phase 3, randomised, open-label CROWN study** | **Lorlatinib versus Crizotinib** | Overlap with Shaw AT, 2020 |
| ***Solomon BJ, 2024*** | **Lorlatinib Versus Crizotinib in Patients With Advanced ALK-Positive Non-Small Cell Lung Cancer: 5-Year Outcomes From the Phase III CROWN Study** | **Lorlatinib versus Crizotinib** | Overlap with Shaw AT, 2020 |
| ***Solomon BJ, 2022*** | **Post Hoc Analysis of Lorlatinib Intracranial Efficacy and Safety in Patients With ALK-Positive Advanced Non-Small-Cell Lung Cancer From the Phase III CROWN Study** | **Lorlatinib versus Crizotinib** | Overlap with Shaw AT, 2020 |
| ***Mazieres J, 2022*** | **Patient-reported outcomes from the randomized phase 3 CROWN study of first-line lorlatinib versus crizotinib in advanced ALK-positive non-small cell lung cancer** | **Lorlatinib versus Crizotinib** | Overlap with Shaw AT, 2020 |
| ***Liu G, 2023*** | **Podcast on Lorlatinib as a First-Line Treatment Option for Patients with ALK-Positive Metastatic NSCLC with Brain Metastasis** | **Lorlatinib versus Crizotinib** | Overlap with Shaw AT, 2020 |
| ***Zhou Q, 2023*** | **Asian Subgroup Analysis of the Randomized Phase 3 CROWN Study of First-Line Lorlatinib Versus Crizotinib in Advanced ALK-Positive NSCLC** | **Lorlatinib versus Crizotinib** | Overlap with Shaw AT, 2020 |
| ***Shaw AT, 2019*** | **Lorlatinib in advanced ROS1-positive non-small-cell lung**  **cancer: a multicentre, open-label, single-arm, phase 1–2 trial** | **Lorlatinib** | Overlap with Benjamin J Solomon,2018 |
| ***Alice T Shaw, 2017*** | **Lorlatinib in non-small-cell lung cancer with *ALK* or *ROS1* rearrangement: an international,multicentre,**  **open-label,single-arm first-in-man phase 1 trial** | **Lorlatinib** | Overlap with Benjamin J Solomon,2018 |
| ***Ross A. Soo,2022*** | **Efficacy and safety of lorlatinib in Asian and non-Asian patients with ALK-positive advanced non-small cell lung cancer: Subgroup analysis of a global phase 2 trial** | **Lorlatinib** | Overlap with Benjamin J Solomon,2018 |
| ***E. Felip,2021*** | **Intracranial and extracranial efficacy of lorlatinib in patients with ALK-positive non-small-cell lung cancer previously treated with second-generation ALK TKIs** | **Lorlatinib** | Overlap with Benjamin J Solomon,2018 |
| ***Rafal Dziadziuszko,2021*** | **Updated Integrated Analysis of the Efficacy and**  **Safety of Entrectinib in Locally Advanced or Metastatic ROS1 Fusion–Positive Non–Small-Cell Lung Cancer** | **Entrectinib** | Overlap with Drilon A,2017 |
| ***NCT01639001*** | **A Study Of Crizotinib Versus Chemotherapy In Previously Untreated ALK Positive East Asian Non-Small Cell Lung Cancer Patients** | **Crizotinib vs** C**hemotherapy** | Overlap with Wu YL, 2018 |
| ***NCT00932893*** | **An Investigational Drug, PF-02341066 Is Being Studied Versus Standard Of Care In Patients With Advanced Non-Small Cell Lung Cancer With A Specific Gene Profile Involving The Anaplastic Lymphoma Kinase (ALK) Gene** | **Crizotinib vs** C**hemotherapy** | Overlap with Blackhall F, 2014 |
| ***NCT01154140*** | **A Clinical Trial Testing The Efficacy Of Crizotinib Versus Standard Chemotherapy Pemetrexed Plus Cisplatin Or Carboplatin In Patients With ALK Positive Non Squamous Cancer Of The Lung (PROFILE 1014)** | **Crizotinib vs** C**hemotherapy** | Overlap with Solomon BJ, 2018 |
| ***NCT01828112*** | **LDK378 Versus Chemotherapy in ALK Rearranged (ALK Positive) Patients Previously Treated With Chemotherapy (Platinum Doublet) and Crizotinib** | **Ceritinib vs Chemotherapy** | Overlap with Shaw AT, 2017 |
| ***NCT01828099*** | **LDK378 Versus Chemotherapy in Previously Untreated Patients With ALK Rearranged Non-small Cell Lung Cancer** | **Ceritinib vs Chemotherapy** | Overlap with Soria JC, 2017 |
| ***NCT03052608*** | **A Study Of Lorlatinib Versus Crizotinib In First Line Treatment Of Patients With ALK-Positive NSCLC** | **Lorlatinib versus Crizotinib** | Overlap with Shaw AT, 2020 |
| ***NCT04632758*** | **Study Comparing WX-0593 to Crizotinib in ALK Positive Non-Small Cell Lung Cancer (NSCLC) Patients** | **Iruplinalkib**  **versus Crizotinib** | Overlap with Shi Y, 2024 |
| ***NCT00932451*** | **An Investigational Drug, PF-02341066, Is Being Studied In Patients With Advanced Non-Small Cell Lung Cancer With A Specific Gene Profile Involving The Anaplastic Lymphoma Kinase (ALK) Gene** | **Crizotinib** | Overlap with Blackhall F, 2017 |
| ***NCT00585195*** | **A Study Of Oral PF-02341066, A C-Met/Hepatocyte Growth Factor Tyrosine Kinase Inhibitor, In Patients With Advanced Cancer (PROFILE 1001)** | **Crizotinib** | Overlap with Shaw AT, 2019 |
| ***NCT01945021*** | **Phase II Safety and Efficacy Study of Crizotinib in East Asian Patients With ROS1 Positive, ALK Negative Advanced NSCLC** | **Crizotinib** | Overlap with Shaw Wu YL,2022 |
| ***NCT01685060*** | **LDK378 in Adult Patients With ALK-activated NSCLC Previously Treated With Chemotherapy and Crizotinib** | **Ceritinib** | Overlap with Shaw Crinò L,2016 |
| ***NCT01685138*** | **LDK378 in Crizotinib naïve Adult Patients With ALK-activated Non-small Cell Lung Cancer** | **Ceritinib** | Overlap with Shaw Nishio M,2020 |
| ***NCT02450903*** | **LDK378 in Patients With ALK Positive NSCLC Previously Treated With Alectinib** | **Ceritinib** | Overlap with Shaw Hida T,2018 |
| ***NCT01970865*** | **A Study Of PF-06463922 An ALK/ROS1 Inhibitor In Patients With Advanced Non Small Cell Lung Cancer With Specific Molecular Alterations** | **Lorlatinib** | Overlap with Benjamin J Solomon,2018 |
| ***NCT03909971*** | **A Study of Lorlatinib in ALK Inhibitor-Treated ALK-Positive NSCLC in China** | **Lorlatinib** | Overlap with Shaw Lu S,2022 |

Table S3. The risk of bias assessments of single-arm studies via ROBINS-I V2 tool.

| Items for assessment | Confounding bias | Subject selection bias | Intervention classification bias | Intention-to-intervention bias | Missing data bias | Outcome measurement bias | Selective reporting bias |
| --- | --- | --- | --- | --- | --- | --- | --- |
| PROFILE 1005, 2017 | Low | Low | Low | Moderate | Low | Low | Low |
| Wu YL, 2022 | Low | Low | Low | Moderate | Low | Low | Low |
| ASCEND-2, 2016 | Low | Low | Low | Low | Moderate | Low | Low |
| ASCEND-3, 2019 | Moderate | Low | Low | Low | Moderate | Low | Low |
| ASCEND-9, 2018 | Moderate | Low | Low | Moderate | Moderate | Low | Low |
| NCT02040870 | Low | Low | Low | Low | Serious | Low | Low |
| Lim SM, 2017 | Low | Low | Low | Moderate | Serious | Low | Low |
| Solomon BJ, 2018 | Moderate | Low | Low | Low | Low | Low | Low |
| Lu S, 2022 | Low | Low | Low | Low | Moderate | Low | Low |
| Seto T, 2020 | Low | Low | Low | Moderate | Low | Low | Low |
| STARTRK-2, 2021 | Moderate | Low | Low | Low | Low | Low | Low |
| TRIDENT-1, 2021 | Moderate | Low | Low | Low | Low | Low | Low |
| TRUST-I, 2024 | Low | Low | Low | Moderate | Moderate | Low | Low |
| INTELLECT, 2023 | Moderate | Low | Low | Low | Moderate | Low | Low |
| Lu S, 2023 | Moderate | Low | Low | Moderate | Low | Low | Low |

In ROBINS-I V2 tool, bias was classified into seven domains: confounding bias; Subject selection bias; intervention classification bias; Intentional intervention bias; missing data bias; Outcome measurement bias; Selective reporting bias. And primarily assessed the risk of bias as low, moderate, serious, critical, or lacking information.

Table S4. The subgroup estimates for gender, ethnicity, age, study type, publication date.

|  |  |  | Incidence (%)  [95% CI] | Heterogeneity | | | |
| --- | --- | --- | --- | --- | --- | --- | --- |
|  | Subgroup | n |  | *I²* | *Z* | *P* | *P* (Heterogeneity between subgroups) |
| AE | Gender |  |  |  |  |  |  |
|  | Female ≤ 55% | 455 | 99 [97-100] | 68.08 | 33.54 | 0.020 | 0.414 |
|  | Female ＞ 55% | 1919 | 98 [96-99] | 70.47 | 57.17 | ＜.001 |  |
|  | Ethnicity |  |  |  |  |  |  |
|  | Asian | 211 | 100 [99-100] | - | 42.81 | -- | 0.003 |
|  | Pan-racial | 1849 | 98 [96-99] | 44.20 | 75.00 | 0.100 |  |
|  | Age |  |  |  |  |  |  |
|  | Age ≤ 54 years | 2085 | 98 [97-99] | 67.39 | 62.70 | ＜.001 | 0.077 |
|  | Age ＞ 54 years | 289 | 100 [98-100] | - | 49.35 | - |  |
|  | Study type |  |  |  |  |  | 0.279 |
|  | RCT | 1212 | 99 [97-100] | 72.95 | 50.63 | ＜.001 |  |
|  | Single-arm | 1162 | 98 [97-98] | - | 94.94 | - |  |
|  | Publication date |  |  |  |  |  | 0.775 |
|  | Publication before (including) 2018 | 1623 | 98 [96-100] | 80.50 | 40.80 | ＜.001 |  |
|  | Publication after 2018 | 751 | 99 [97-100] | 53.69 | 51.89 | 0.060 |  |
| SAE | Gender |  |  |  |  |  |  |
|  | Female ≤ 55% | 206 | 42 [32-53] | 81.54 | 11.94 | ＜.001 | 0.946 |
|  | Female ＞ 55% | 911 | 43 [34-52] | 91.70 | 15.12 | ＜.001 |  |
|  | Ethnicity |  |  |  |  |  |  |
|  | Asian | 98 | 46 [39-53] | - | 20.22 | - | 0.521 |
|  | Pan-racial | 877 | 42 [34-52] | 91.71 | 14.51 | ＜.001 |  |
|  | Age |  |  |  |  |  |  |
|  | Age ≤ 54 years | 996 | 43 [36-50] | 87.28 | 19.55 | ＜.001 | 0.682 |
|  | Age ＞ 54 years | 121 | 41 [36-47] | - | 22.43 | - |  |
|  | Study type |  |  |  |  |  |  |
|  | RCT | 494 | 39 [33-45] | 79.91 | 19.77 | ＜.001 | 0.000 |
|  | Single-arm | 623 | 52 [39-55] | - | 53.54 | - |  |
|  | Publication date |  |  |  |  |  | 0.979 |
|  | Publication before (including) 2018 | 781 | 43 [35-50] | 85.01 | 17.46 | ＜.001 |  |
|  | Publication after 2018 | 336 | 43 [31-55] | 91.70 | 10.52 | ＜.001 |  |


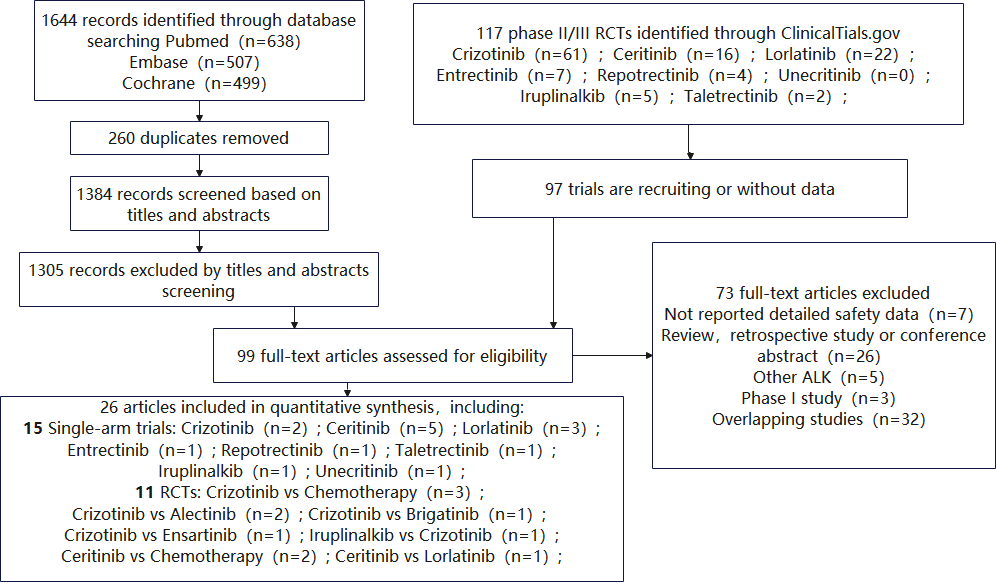


**Figure S1.** Flow diagram for the selection of eligible studies. RCT: random controlled trials.


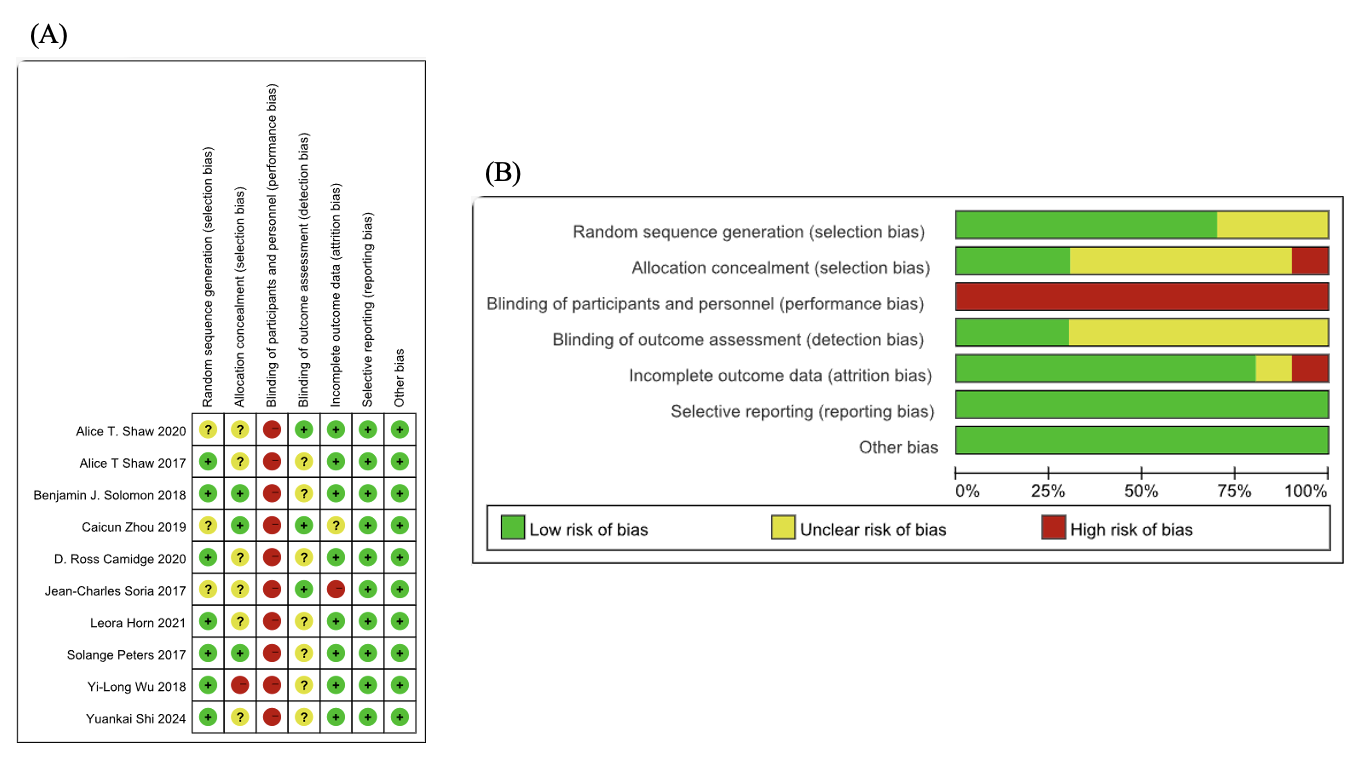


**Figure S2.** The risk of bias assessments of RCTs via Cochrane Risk of Bias Tool. (A) Risk of bias summary: judgements about each risk of bias item for each included study. (B) Risk of bias graph: judgements (low, unclear and high) about each risk of bias item presented as percentages across all included studies.

(A)


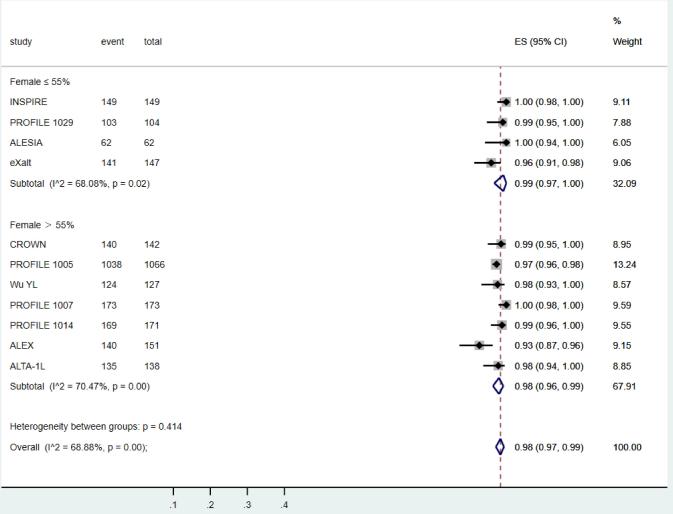


(B)


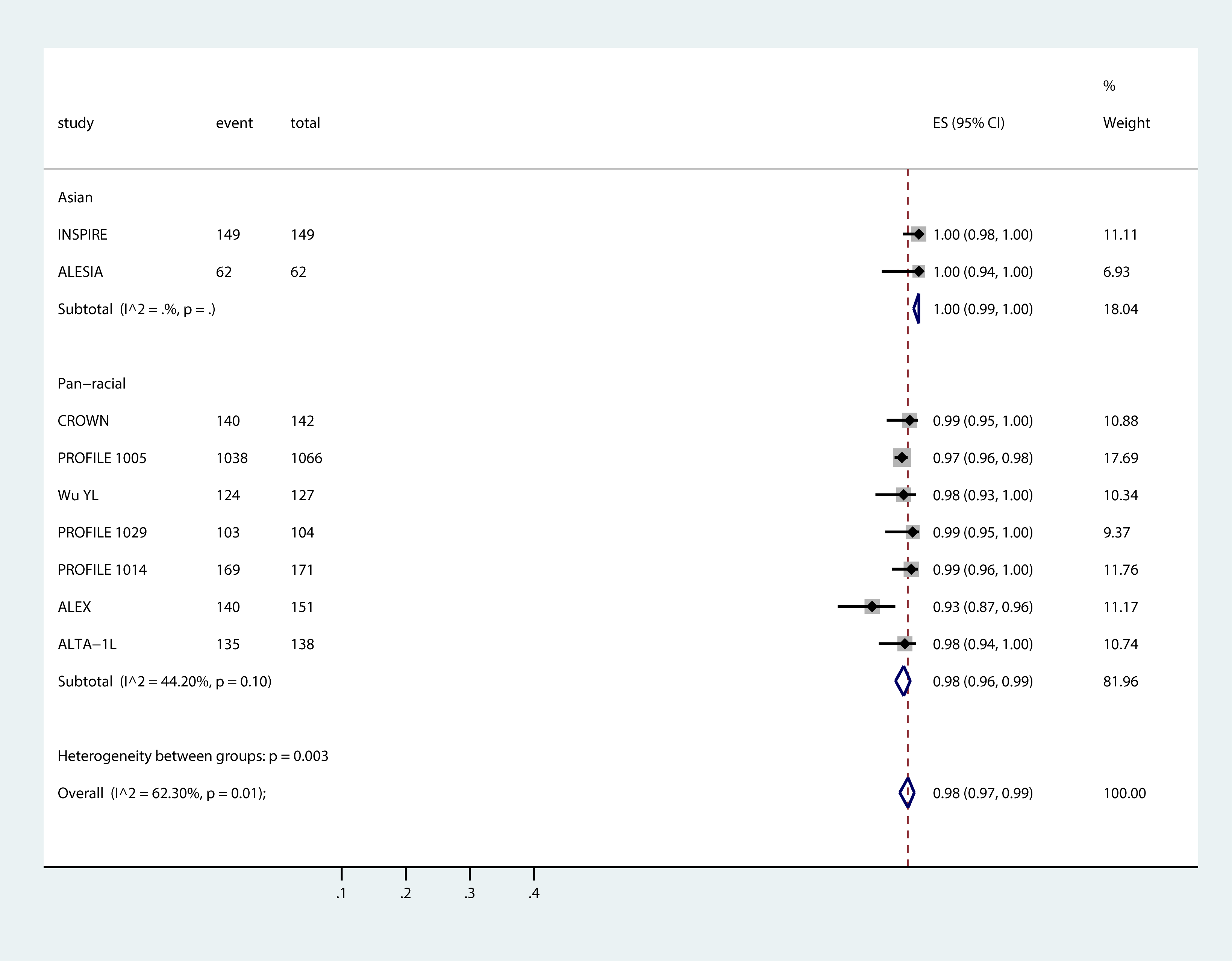


(C)


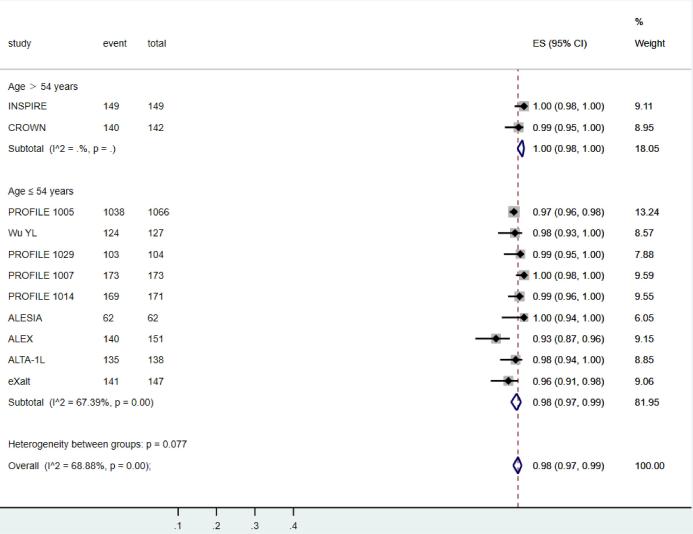


(D)


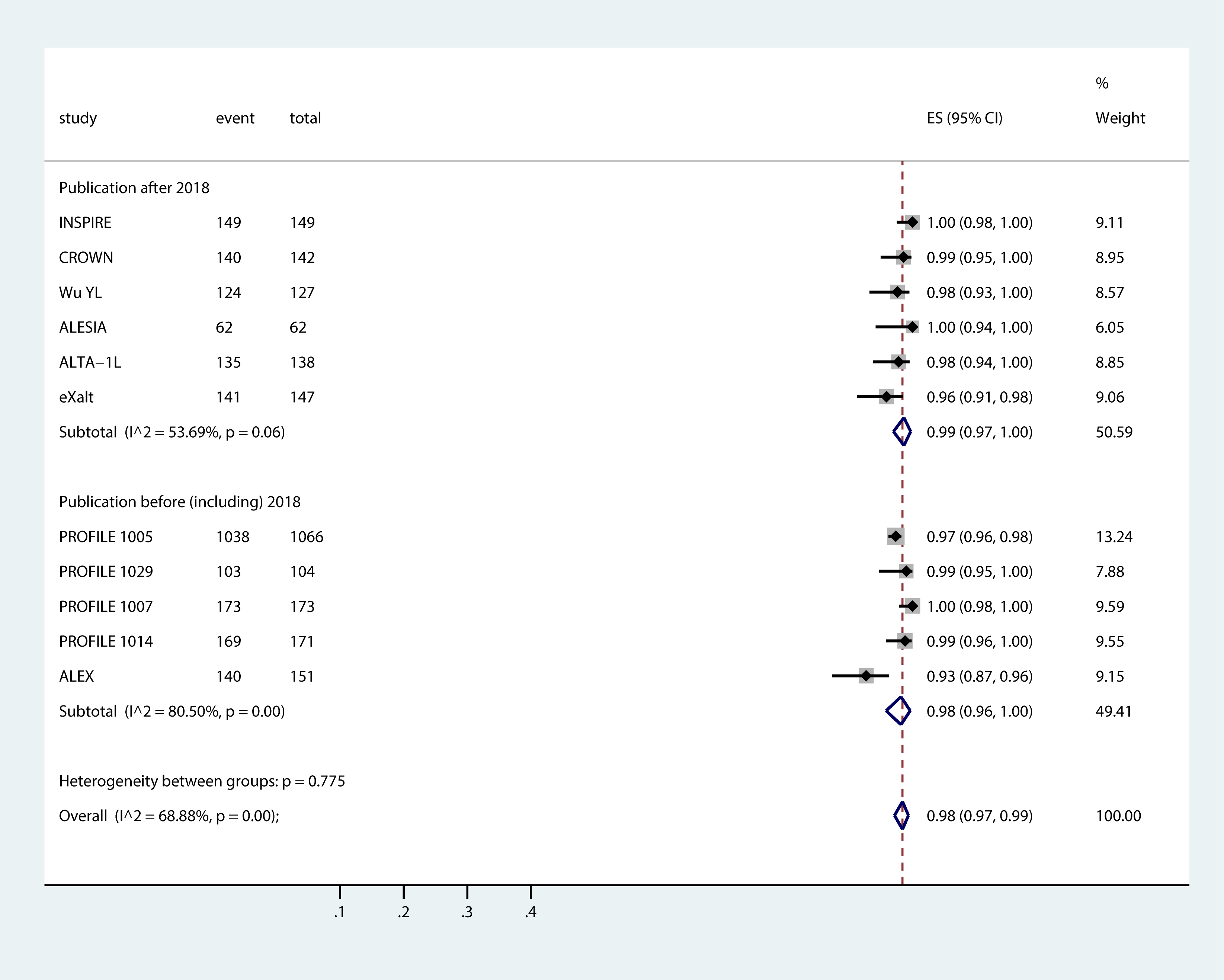


(E)


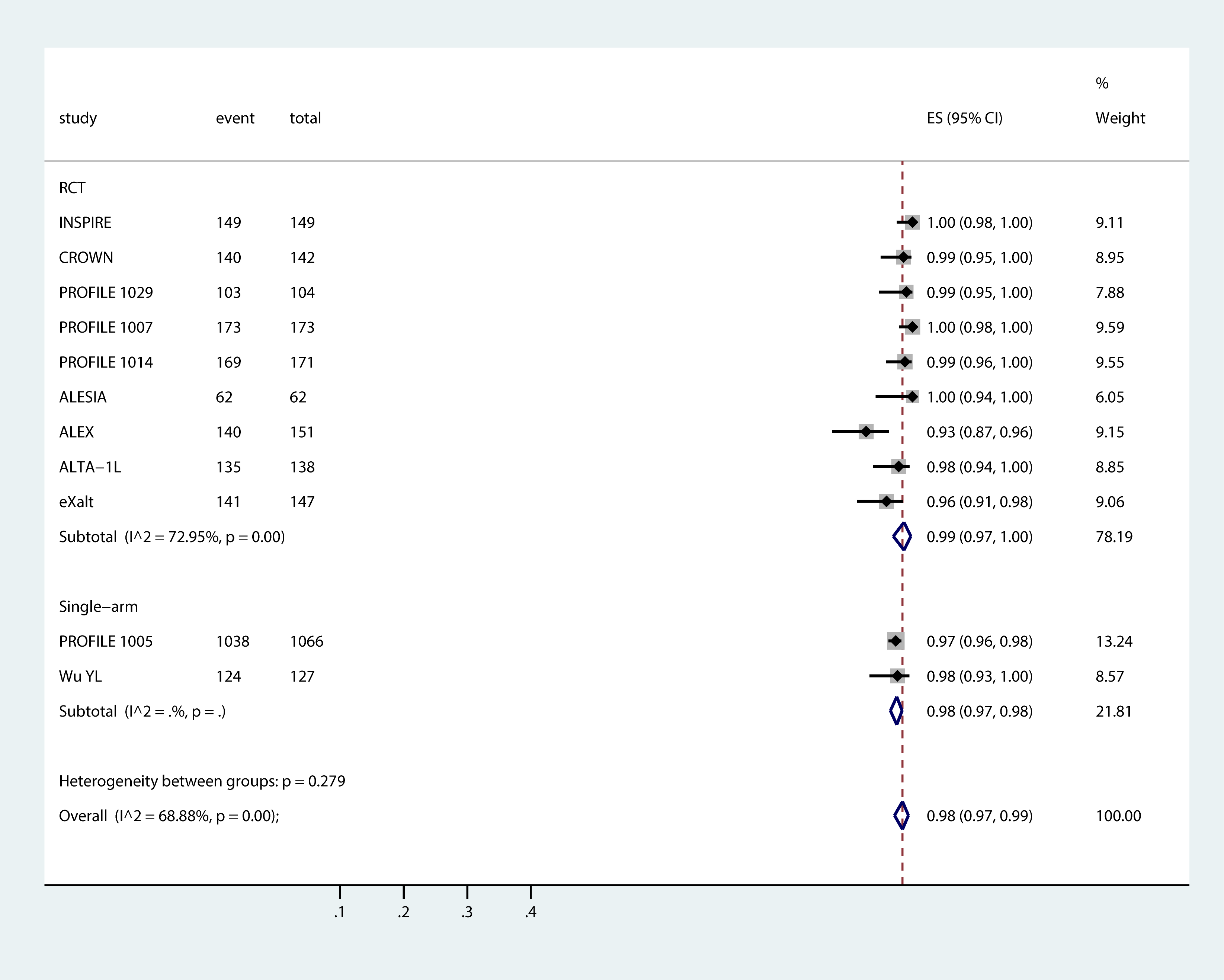


**Figure S3.** Forest plots of subgroup analysis of heterogeneity in the incidence of systemic all-grade AEs associated with Crizotinib based on variables such as gender (A), ethnicity (B), age (C), publication date (D), study type (E).

(A)


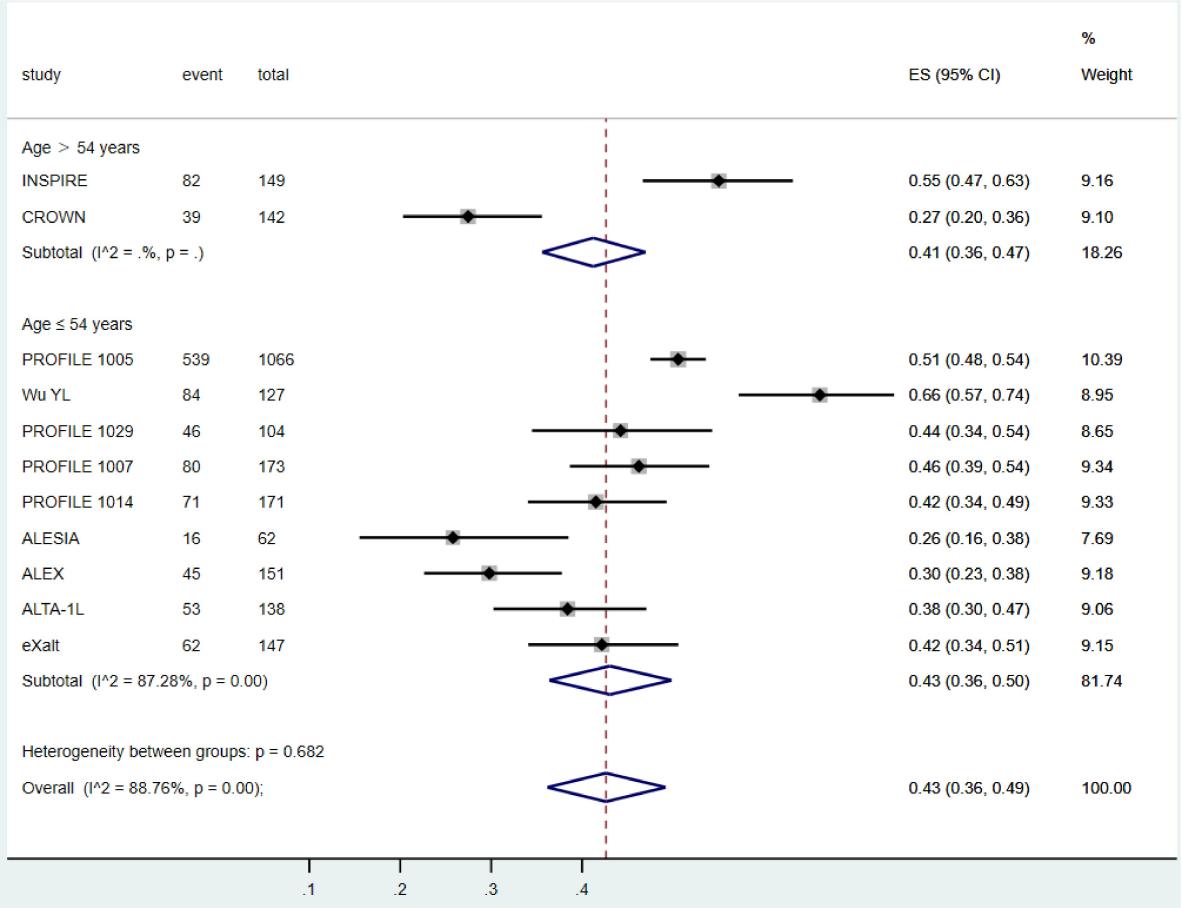


(B)


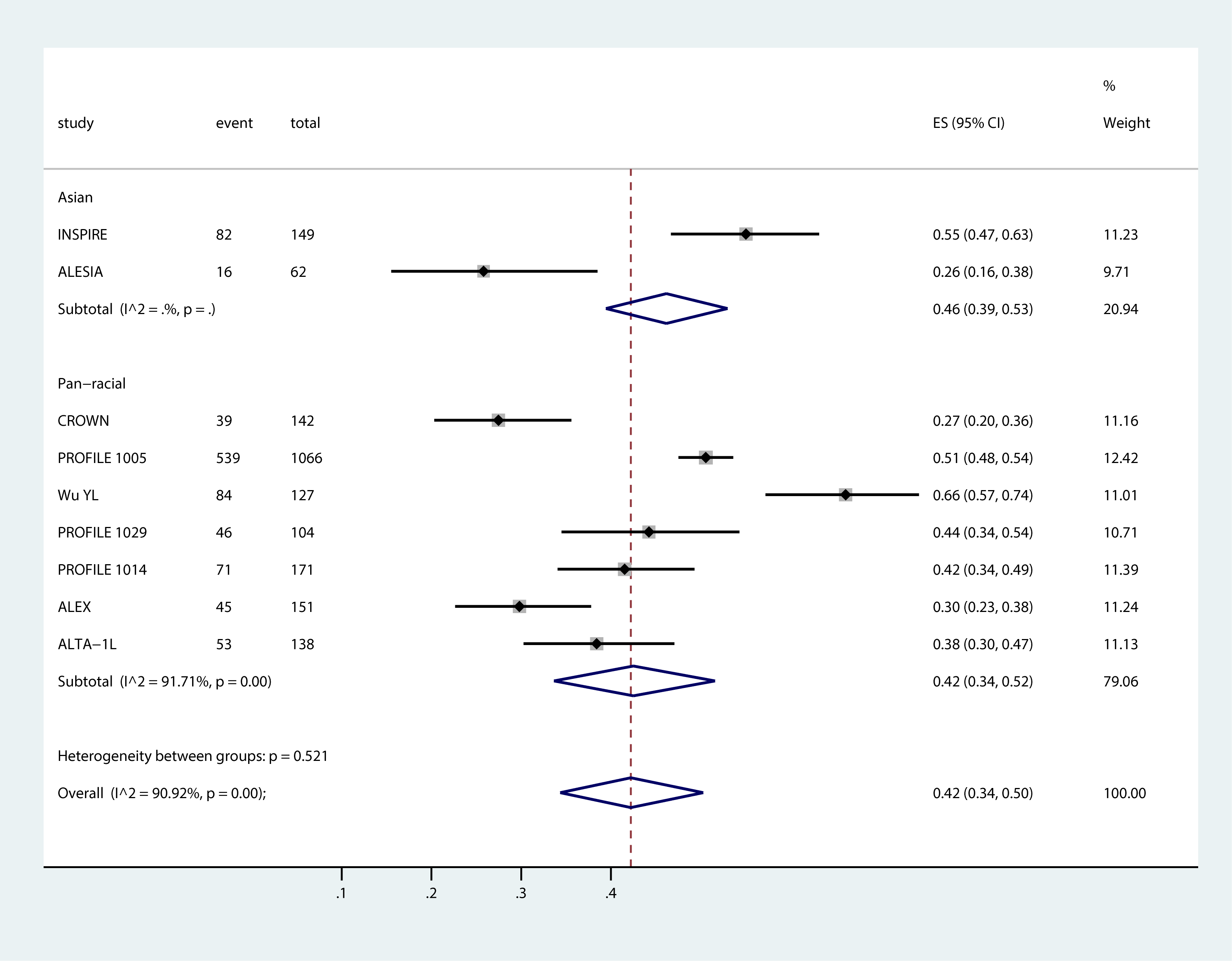


(C)


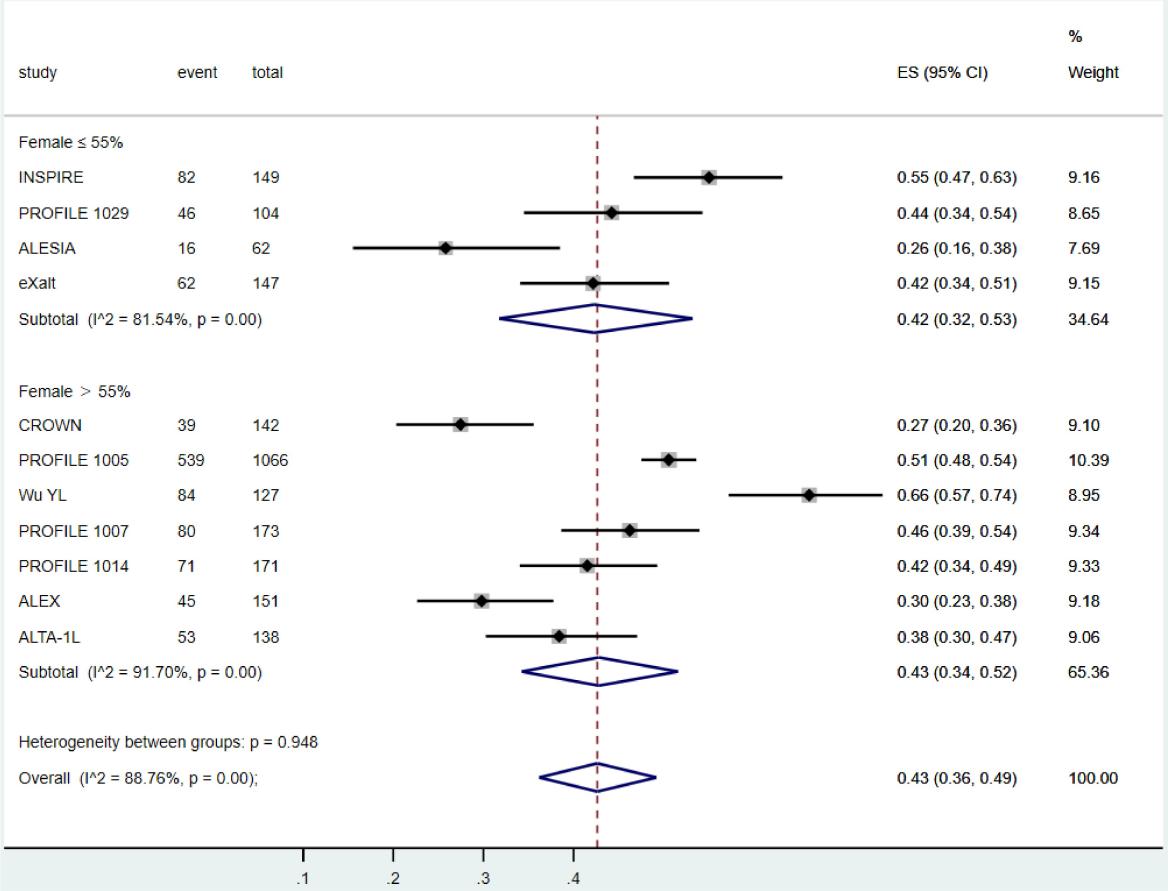


(D)


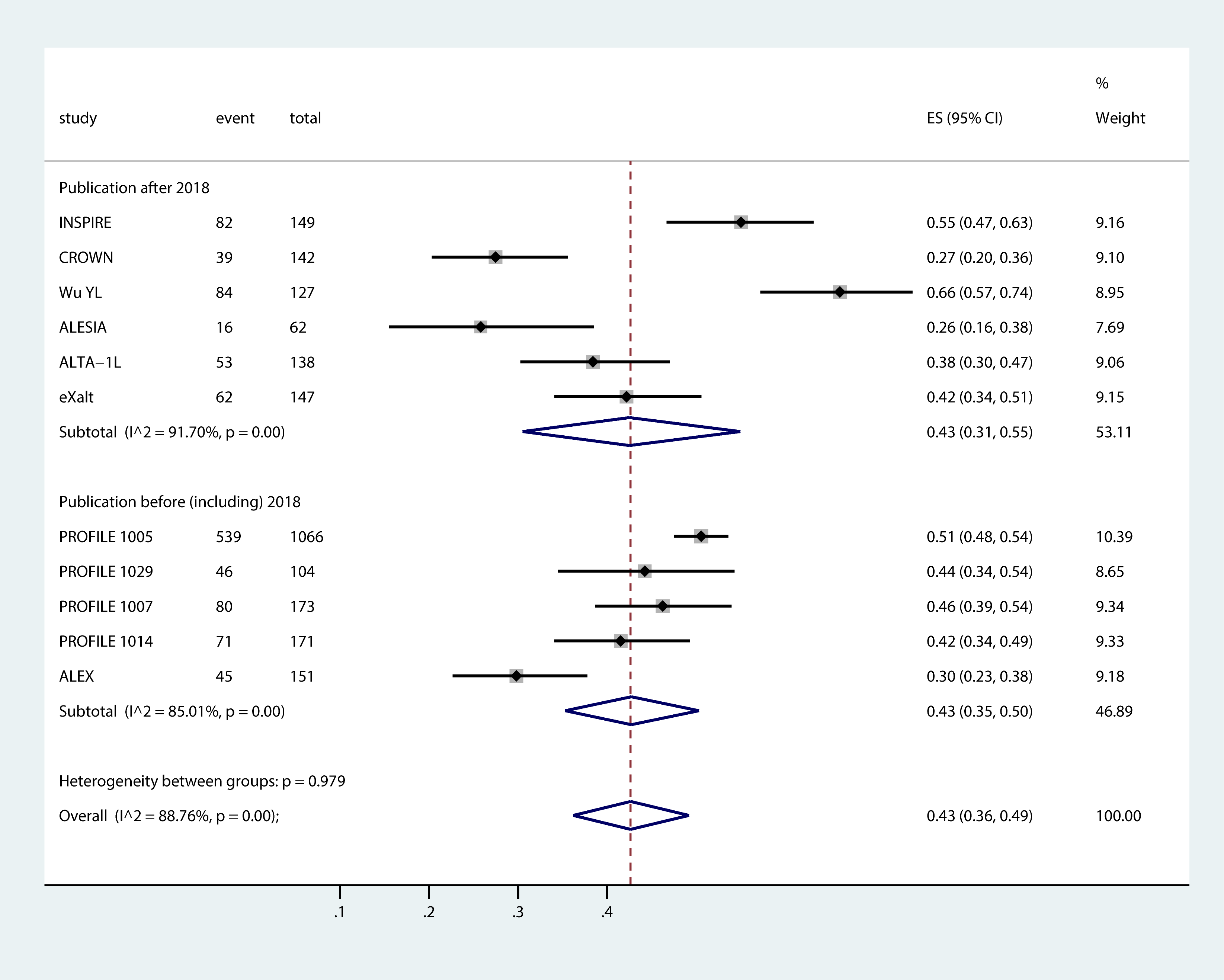


(E)


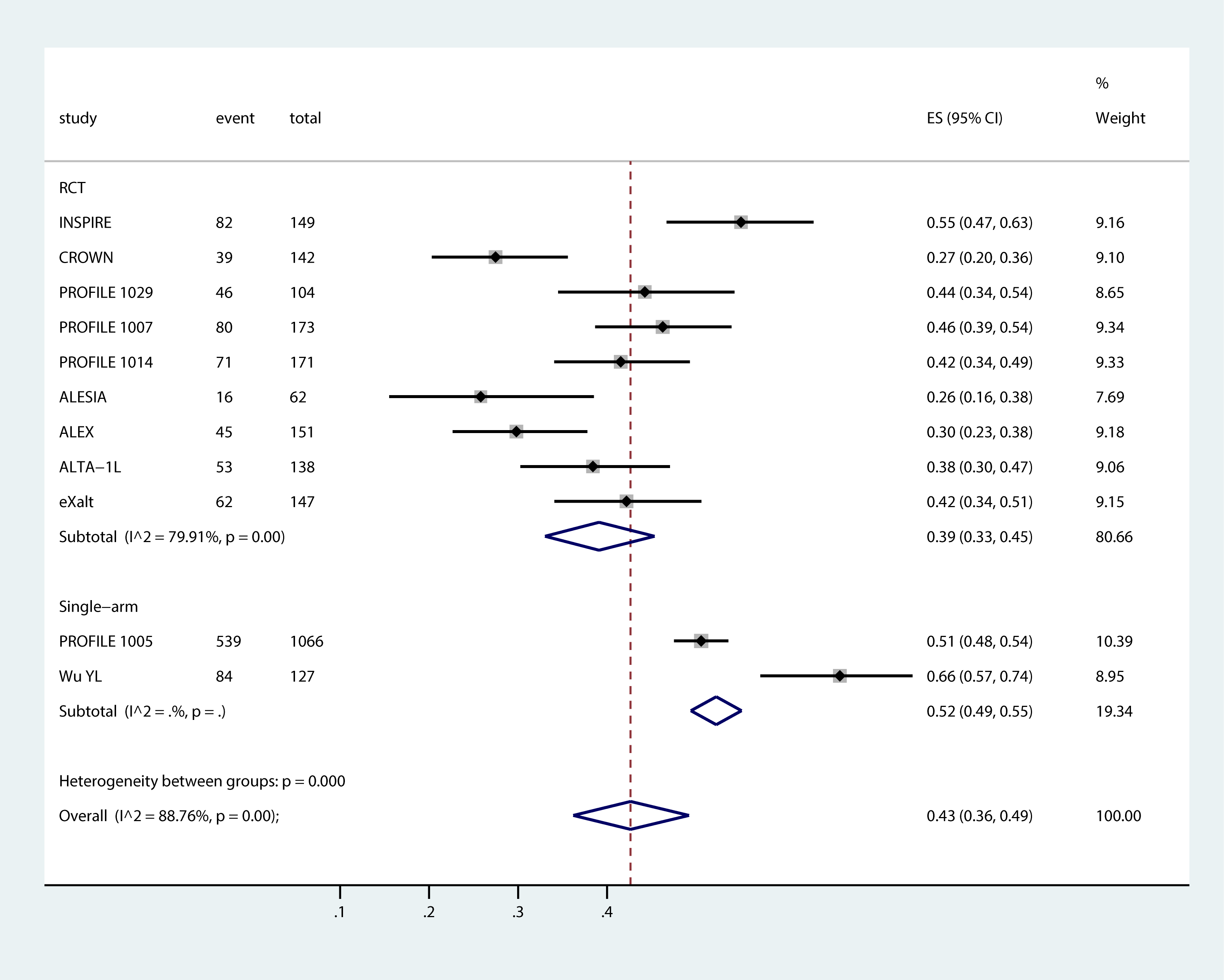


**Figure S4.** Forest plots of subgroup analysis of heterogeneity in the incidence of systemic SAEs associated with Crizotinib based on variables such as gender (A), ethnicity (B), age (C), publication date (D), study type (E).

(A)


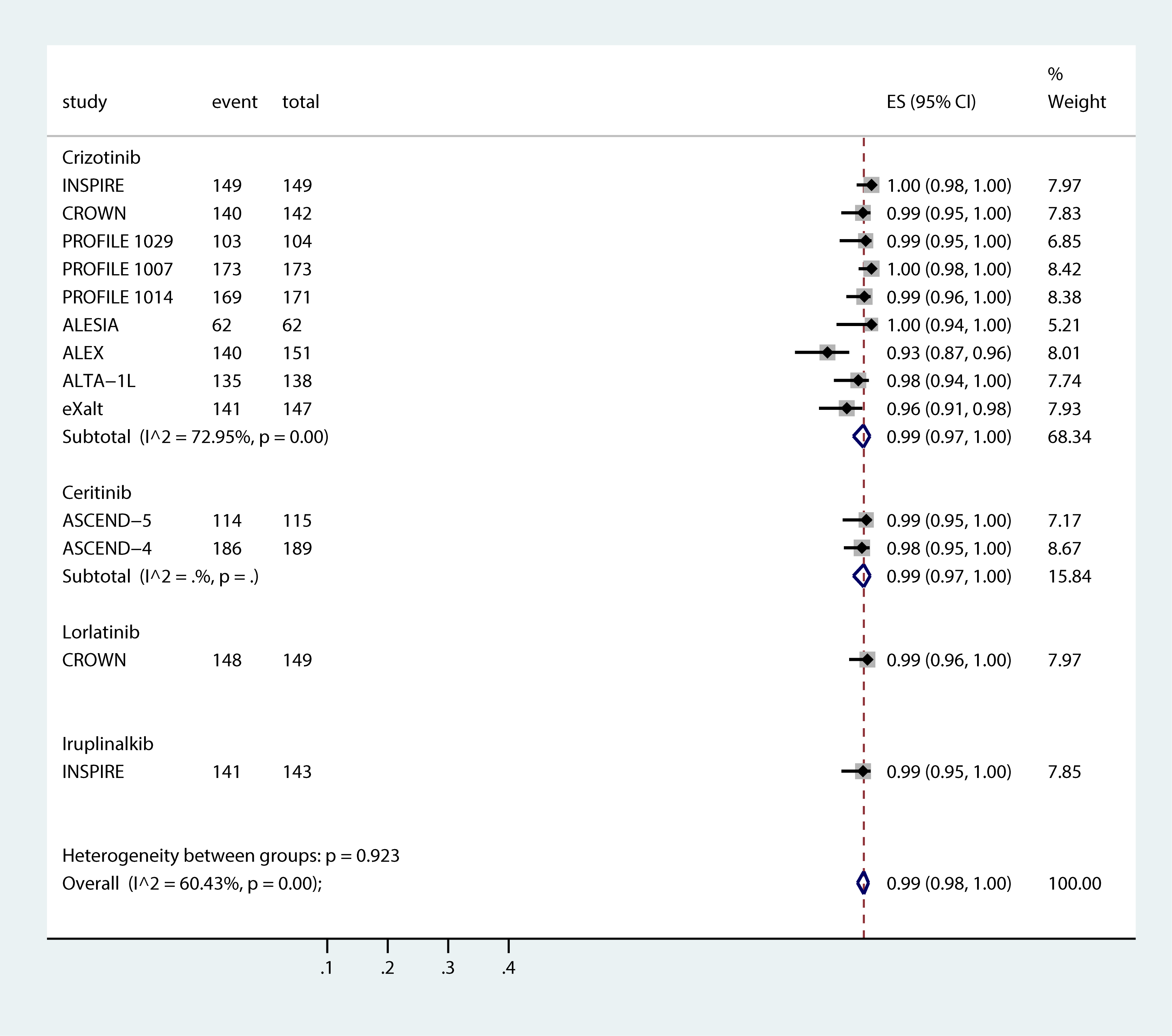


(B)


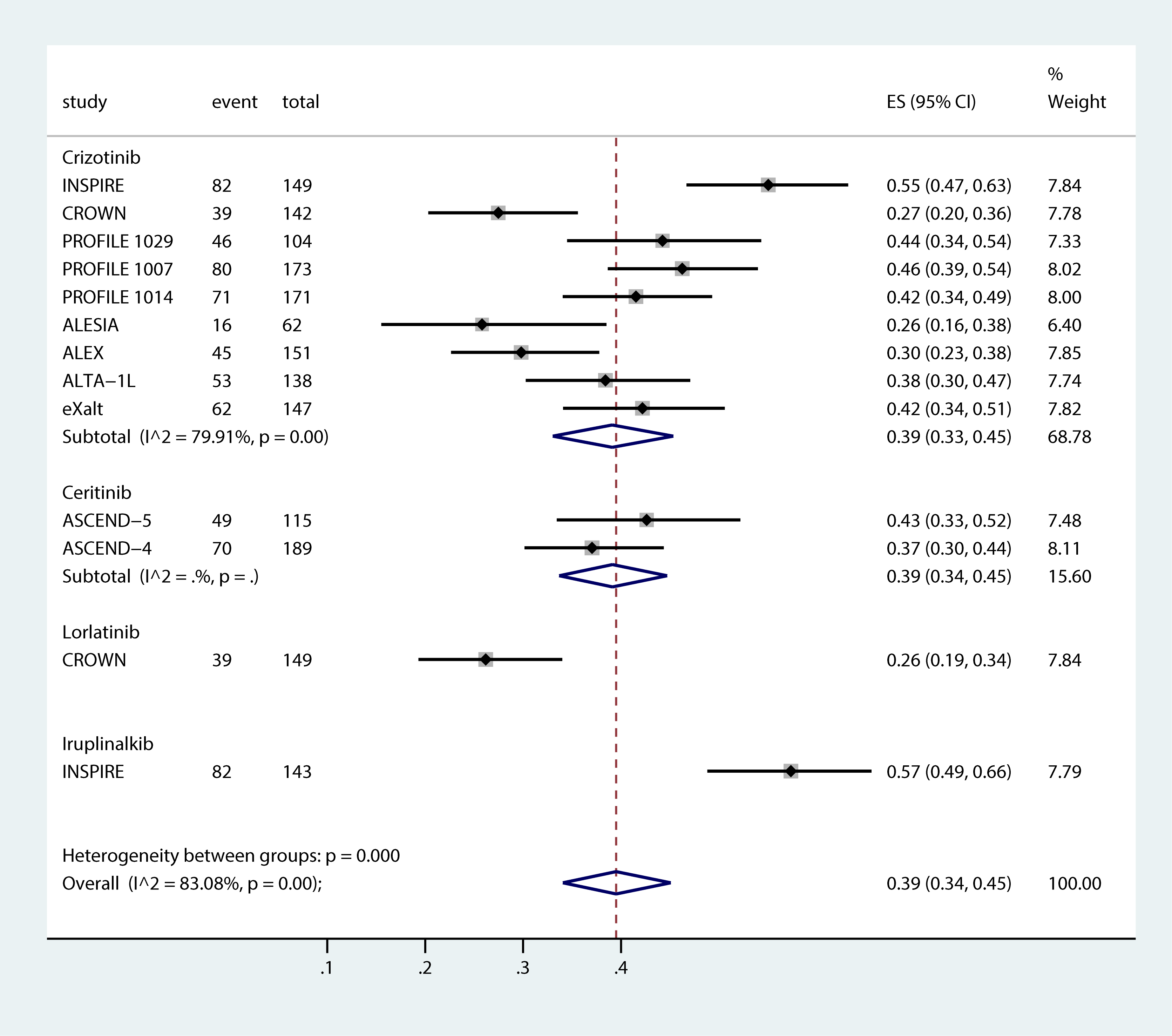


**Figure S5.** Forest plots of incidences of all-grade AEs (A) and SAEs (B) restricting RCTs.
